# Effects of Wintering Environment and Parasite–Pathogen Interactions on Honey Bee Colony Loss in North Temperate Regions

**DOI:** 10.1371/journal.pone.0159615

**Published:** 2016-07-22

**Authors:** Suresh D. Desai, Robert W. Currie

**Affiliations:** Department of Entomology, University of Manitoba, Winnipeg, Manitoba, R3T 2N2, Canada; Columbia University, UNITED STATES

## Abstract

Extreme winter losses of honey bee colonies are a major threat to beekeeping but the combinations of factors underlying colony loss remain debatable. We monitored colonies in two environments (colonies wintered indoors or outdoors) and characterized the effects of two parasitic mites, seven viruses, and *Nosema* on honey bee colony mortality and population loss over winter. Samples were collected from two locations within hives in fall, mid-winter and spring of 2009/2010. Although fall parasite and pathogen loads were similar in outdoor and indoor-wintered colonies, the outdoor-wintered colonies had greater relative reductions in bee population score over winter. Seasonal patterns in deformed wing virus (DWV), black queen cell virus (BQCV), and *Nosema* level also differed with the wintering environment. DWV and *Nosema* levels decreased over winter for indoor-wintered colonies but BQCV did not. Both BQCV and *Nosema* concentration increased over winter in outdoor-wintered colonies. The mean abundance of *Varroa* decreased and concentration of Sacbrood virus (SBV), Kashmir bee virus (KBV), and Chronic bee paralysis virus (CBPV) increased over winter but seasonal patterns were not affected by wintering method. For most viruses, either entrance or brood area samples were reasonable predictors of colony virus load but there were significant season*sample location interactions for *Nosema* and BQCV, indicating that care must be taken when selecting samples from a single location. For *Nosema* spp., the fall entrance samples were better predictors of future infestation levels than were fall brood area samples. For indoor-wintered colonies, Israeli acute paralysis virus IAPV concentration was negatively correlated with spring population size. For outdoor-wintered hives, spring *Varroa* abundance and DWV concentration were positively correlated with bee loss and negatively correlated with spring population size. Multivariate analyses for fall collected samples indicated higher DWV was associated with colony death as did high SBV for spring-collected samples.

## Introduction

The beekeeping, and pollination industries worldwide are greatly affected by the recent challenges to managed honey bee (*Apis mellifera* L.) colonies resulting winter losses of honey bee colonies often averaging 30–40% in the U.S., Canada and Europe [1–4]. These losses are generally believed to result from interactions with multiple stressors that include parasitic mites, pathogens (viruses, bacteria, and microsporidia parasitic fungi), poor queen quality, low genetic diversity, pesticides and other environmental factors [1, 3, 5].

Honey bee viruses are wide spread in Canada and some, such as deformed wing virus (DWV) and black queen cell virus (BQCV), are present in most colonies–often at high concentrations [6]. However, the roles that bee viruses and their interactions with other parasites, pathogens and environmental stressors play in contributing to winter colony losses are unclear. It is important to determine which of these pathogens, or groups of parasites and pathogens, has the greatest impact on winter mortality of honey bee colonies, under different wintering management (indoor and outdoor wintering) conditions. This information would assist in the development of effective management strategies.

The ectoparasitic mite, *Varroa destructor* Anderson and Trueman causes winter loss of colonies when mite levels are greater than 10% in late fall [7–9] and is a significant cause of winter losses of honey bee colonies in the northern hemisphere [2, 10–12]. *Varroa* can have synergistic interactions with other parasites such as the tracheal mite, *Acarapis woodi* (Rennie), resulting in high winter loss even with low levels of *Varroa* present [8, 13, 14]. Multiple infestations of honey bee colonies with a variety of microbes might also play a role in winter colony mortality [3, 5, 15–17]. Viruses directly play a role in affecting bee colony population loss that is equivalent to, or larger than, direct *Varroa* feeding damage [18]. However, little is known about interactional effects between *Varroa* and pathogens of honey bees or how beekeepers can manage colonies to decrease the impact of such interactions in overwintering colonies. Three viruses in particular, (DWV, Israeli acute paralysis virus (IAPV) and acute bee paralysis virus (ABPV)) have been linked with large scale overwinter losses [15, 16, 19–21]. ABPV is suspected to be involved in colony losses in Europe [10, 21, 22] but its role in colony loss in North America is less clear. Other viruses of importance include KBV, BQCV, SBV, and CBPV. Links between KBV and colony loss occur. For example, prevalence of KBV in CCD colonies is greater than in non-CCD colonies [20, 23]. BQCV is closely associated with the microsporidian *Nosema apis* Z. and may work in concert with it to affect honey bee health but it is not thought to be a major factor in colony collapse in some areas of Europe [15]. CBPV and SBV were the second most prevalent viruses identified in a study conducted in Belgium but neither were correlated with colony mortality[22].

One of the biggest challenges of studying virus pathogenesis in honey bees is linking infection by an individual virus to a particular set of economic impacts or disease symptoms. In field studies, honey bees are often infected by multiple viruses simultaneously, most of which usually persist as latent infections in the bee hosts [24]. In addition, virus infections in honey bees are often associated with non-viral pathogens and other parasites. Therefore, without the application of Koch’s postulates, it is difficult to prove that specific symptoms are indeed caused by a particular virus and not the result of mixed virus infections particularly when viral loads in bees cannot be determined. Virus prevalence information is not adequate to predict colony loss [6] so quantification of virus loads using sequence-based methods is essential to developing estimates of the potential impacts of individual viruses on infected bees [25]. Information as to how and when samples should be collected in order to best predict the disease impact of viruses is also required.

The other groups of parasites associated with colony losses in temperate countries are the microsporidian fungi, *N*. *apis* and *N*. *ceranae* (mentioned previously). Nosema has long been known as an economically important and commonly encountered disease [26, 27]. *Nosema ceranae* is associated with reduced honey production and increased winter mortality [28, 29]. A nominal treatment threshold of 1 million spores per bee is recommended in Canada [30], but this estimate was based upon *N*. *apis* and good thresholds do not exist for *N*. *ceranae*.

The level of *Nosema* is typically higher in foragers or old worker bees than newly emerged bees and house bees [31] suggesting older workers may be better indicators of future disease impacts. Methods of sampling for *Nosema* may need to be refined to better predict its impacts and to develop thresholds for this organism [32]. Similar issues related to sampling of bees need to be addressed with respect to both viruses and *Varroa*. Thus, studies on the best location in the hive for collecting samples for these groups of parasites and pathogens are required. Long term monitoring of indoor and outdoor-wintered colonies prior to symptoms of collapse is required to help identify the pathogens associated with colony losses.

Little is known about the effects of the winter environment on virus interactions. This can be examined in Canada where there are two primary types of wintering methods adopted by the beekeepers. Honey bee colonies can be wintered indoors in an environmentally controlled building, or outdoors, protected by insulation. For indoor-wintering, honey bee colonies are stored in a building under complete darkness where temperatures are maintained at about 2°C– 5°C. Beekeepers may over winter honey bee colonies in a single brood chamber or multiple brood chambers. In indoor-wintering method, a majority of beekeepers in western Canada winter bee colonies in single brood-chamber hives whereas for outdoor wintering, the majority winter bee colonies in double brood chamber hives. Differences in susceptibility to *Varroa*, combinations of *Varroa* and tracheal mite and possibly *Nosema* may occur in colonies wintered indoors and those wintered outdoors and susceptibility to viruses may be similarly affected [8, 14, 33–37].

The overall purpose of this study was first, to understand the seasonal dynamics and relative importance of parasites and pathogens on winter mortality under indoor and outdoor-wintering management systems and second, to determine if practical sampling methods can be developed to help predict their impact on colony survival over winter. The outcome of this study may help beekeepers, to understand the dynamics of disease and pathogen interactions and thus reduce winter mortality of honey bee colonies or optimize the management of parasites. We show that winter environment affects the dynamics of interactions between parasites, pathogens and colony population losses, and that sample location within the hive can affect interpretation of pathogen load results for some but not all pathogens.

## Materials and Methods

### Apiary and colony selection

Honey bee colonies were sampled from five different beekeeping regions in the Province of Manitoba, Canada (Eastern, Southwest, Northwest, Interlake, and Central) (S1 Fig). Prior permission was obtained from 25 beekeepers to collect samples from their bee yards. The distance between beekeepers was in the range of 30km to 150km within the same region. Five beekeepers that wintered bees using either an outdoor or indoor wintering management system were randomly selected from each region (except for regions in which beekeepers used only one wintering method). For each beekeeper, three colonies were randomly selected from a single apiary site for inclusion in the study. Fifteen of these beekeepers practiced indoor wintering, and 10 of them practiced outdoor wintering. Hence, 45 colonies were wintered using indoor-wintering buildings (33 in single chamber standard Langstroth hives containing 9–10 Hoffman frames and 12 in double chamber hives) (here after referred to as “indoor-wintered”) [38] and 30 colonies were wintered outdoors (9 in single chamber and 21 in double chamber hives) (here after referred to as “outdoor-wintered”). Beekeepers were asked to follow their usual apicultural management techniques for controlling parasites, wintering and managing colonies. In fall, 14 of 15 producers that wintered indoors and 9 of 10 producers that wintered outdoors treated bees to control *Varroa*. Acaricides used were amitraz (Apivar ^®^) (11 producers), formic acid (various formulations) (10 producers), oxalic acid (2 producers), and coumaphos (1 producer). In fall, 11 of 15 producers that wintered indoors and 4 of 10 producers that wintered outdoors treated bees with fumagillin to control *Nosema*.

### Data and sample collection

For each of the 75 colonies, adult population (bee cluster size) was estimated in fall prior to wintering and in spring, when colonies were removed from the wintering building (indoor-wintered) or unwrapped (outdoor-wintered). In order to minimize disturbance to the colony, the honey bee population size was estimated from above and below, by counting the number of frame seams completely covered with bees and multiplying by 2,430 bees [39].

Adult honey bee samples were collected from two locations within each test colony, to assess the levels of *Varroa*, tracheal mites, seven viruses, and *Nosema*: one at the “entrance” (front) of the hive and the other from a frame removed from the “brood area” (inside). In the fall (25 September to 8 October), samples of approximately 250 bees were collected from the brood area inside the hive using 300 ml sample cups (at a time when most beekeepers would have already initiated or completed *Varroa* treatment). Additional samples of approximately 200 bees were collected from the entrance using a modified vacuum pump that sucked foragers and presumed “older” bees from the entrance into a cup. Bees were immediately transferred into 250 ml sample cups with a screened lid and the bees were provided with a sugar cube as a feed source to keep them alive until they could be returned to the lab. All bee samples were stored immediately in a -80°C freezer for further analysis. All colonies (indoor and outdoor) were resampled in spring (07 April to 3 May); only indoor-wintered colonies were resampled in mid-winter (5 January to 15 January), as the beekeepers believed outdoor-wintered hives could not be unwrapped without causing damage to the colony. To collect mid-winter samples in the wintering buildings, the top lids of hives were opened and bee samples (approximately 250 bees) were carefully scooped into 300ml sample cups as quickly as possible to minimize colony disturbance. Spring sampling was done in the months of April and May when the hives came out of the over wintering building and outdoor-wintered colonies were unwrapped. In spring, we again collected adult worker bees, from two locations within the hive (i.e. brood area and entrance), measured the size of the honey bee cluster, counted number of live and dead colonies, and recorded any treatments that had been applied by beekeepers. Bee samples were collected from all surviving colonies. For weaker colonies with less than 300 bees, the surviving bees were sampled and the colony was then marked as dead. Percent change in bee population was calculated by comparing fall and spring population scores.

### Quantification of parasites and pathogens

The mean abundance of *Varroa* (mites per 100 bees) [40] on live bees and on dead bees from colonies that had died was assessed by the alcohol shake method [7]. Tracheal mite prevalence was assessed by the thoracic slice method according to [41] using a subsample of 100 bees. *Nosema* spore mean abundance was assessed using a subsample of 100 bees, according to the methods of [42]. Viruses were quantified as described below.

### RNA extraction

Sub samples of 50 frozen adult bees were crushed in a mortar under liquid nitrogen. The total RNA was extracted using an RNeasy Mini Kit (Qiagen, Valencia, CA, USA) following the manufacturer’s instructions. RNA samples were dissolved in DEPC-treated water in the presence of ribonuclease inhibitor and stored at -80°C for further analysis.

### qPCR analysis

A semi-quantitative analysis (qPCR) was performed to examine the difference in relative virus concentration of seven single stranded RNA viruses (DWV, BQCV, SBV, IAPV, KBV, ABPV, and CBPV). The primers for the qPCR are listed in Table A in S1 file. The assays were performed in 20 μL volumes, containing 1μL cDNA (5 fold dilution), 10 μL SYBR green PCR Master Mix (Applied Biosystems, Foster City, CA, USA), and 0.5 μL of each primer. Reaction volume was adjusted with water. Amplifications were performed in triplicate on an ABI Prism 7300 real time PCR machine (Applied Biosystems, Foster City, CA, USA) with the following PCR conditions a single cycle at 95°C for 5 min, 40 cycles at 95°C for 15 s, 58°C for 30 s. An additional step of 72°C for 30 s was added to measure the dissociation curve and data collection. Non-template controls (reaction mix without template) were included in triplicates in all batches. Honey bee actin was used as an endogenous control gene. Actin allows for the normalization of differences in cDNA reactions. The relative virus concentrations were calculated using the 2^-ΔCt^ method, where Ct indicates the cycle threshold. Relative expression was calculated as 2^-ΔCt^; where ΔCt = Ct _(virus gene)_- Ct _(β-actin)_ [43]. Virus prevalence was expressed as the proportion of colonies with detectable levels of each virus (ABPV, BQCV, CBPV, DWV, IAPV, KBV, and SBV).

### Statistical analysis

Data for bee population size, virus concentration (2^-ΔCt^) values, *Varroa* mean abundance, *Nosema* spore mean abundance, tracheal mite prevalence, and bee population loss over winter for each colony were analyzed as follows. Prior to doing the analysis the following transformations were applied: *Varroa* abundance (mites per 100 bees) and tracheal mite prevalence data were arcsine-transformed, bee population score data were square root + 0.5 transformed and *Nosema* levels were log_10_ transformed [44]. The effect of wintering methods (indoor and outdoor) on the change in size of the colony population between fall and spring was analyzed by PROC MIXED (SAS 1999) using a repeated measures design with hives as subjects and season as a repeated measure using the REML statement (restricted maximum likelihood). Where significant interaction was observed (*P* < 0.05) between treatment factors, differences between means were compared by Bonferroni-corrected contrasts [45].

Proportions of colonies infected with different parasites and pathogens in each season (fall and spring) and wintering method (indoor and outdoor-wintering) were compared using binary logistic regression (PROC CATMOD, SAS 1999). Significant pathogen *season interactions were found so separate analyses were performed on each pathogen. A separate analysis was performed for indoor wintered hives to compare mid-winter samples with fall and spring samples.

The response variables for the seven virus concentrations (2^-ΔCt^), *Varroa* mean abundance, *Nosema* spore mean abundance and tracheal mite prevalence were analyzed using a mixed model ANOVA, PROC MIXED (SAS 1999) using the REML statement (restricted maximum likelihood). Wintering method (indoor and outdoor), sample location (entrance and brood area) and date (fall, winter and spring) were treated as fixed effects. Beekeepers (nested within region) and wintering method and hive (nested within beekeepers), region and wintering method and sample location (nested within beekeeper, region, wintering method and hive) were considered random effects. Data were analysed based on the following model:

$$y\;=\;\mu \;+\;{\text{w}}_{i}\;+\;{\text{s}}_{j}+\;{\text{d}}_{k}\;+\;{\text{r}}_{l}\;+\;{\text{ws}}_{ij}\;+\;{\text{wd}}_{ik}\;+\;{\text{wr}}_{il}\;+\;{\text{sd}}_{jk}\;+\;{\text{wsd}}_{\;ijk}\;+\;{\text{b}}_{\;ilm}\;+\;{\text{h}}_{\;ilmn}\;+\;{\text{sh}}_{ijlmn}\;+\;{\text{e}}_{ijklmn}$$

where w is the effect of wintering method (*i* indexes indoor and outdoor), considered a fixed effect,

s_*j*_ = sample location (*j* indexes entrance and brood area), considered fixed,

d_*k*_ = date (*k* indexes fall, winter and spring), considered fixed,

r_*l*_ = region (*l* indexes Eastern, Southwest, Northwest, Interlake, and Central), considered fixed, two and three-way interactions are denoted by letter combinations of the above main effects,

b _*ilm*_ = the effect of the *m*^th^ beekeeper nested in the *l*^th^ region and *i*^th^ wintering treatment, considered random,

h _*ilmn*_ = the effect of the *n*^th^ hive nested in the *m*^th^ beekeeper and the *l*^th^ region and *i*^th^ wintering treatment, considered random

sh_*ijlmn*_ = the interaction of the jth sampling location with the *n*^th^ hive nested in the *m*^th^ beekeeper and the *l*^th^ region and *i*^th^ wintering treatment, considered random, and

e_ijklmn_ = the residual error

Interactions with region were excluded from the model. Where significant interactions were observed, they were partitioned using the SLICE option in the LSMEANS statement with PROC MIXED by both wintering method and by season to compare differences between means within levels of treatments.

Correlations between bee loss (percent reduction in bee population as measured from fall to spring), colony population in spring and pathogen and parasite levels (as measured both in fall and spring), were analyzed using pooled values for brood area and entrance samples using simple correlations (PROC CORR, SAS Institute Inc., 1999). The partial correlations were calculated using multivariate ANOVA (PROC GLM with option MANOVA, SAS Institute Inc., 1999). The simple and partial correlations were adjusted for multiple testing using a false-discovery rate (FDR) procedure [46, 47]. We calculated the *q*-values using a bootstrap technique with FDR and have included all values that met a cut off filter of 0.20 [48] using the QVALUE R-statistical software package http://genomics.princeton.edu/storeylab/qvalue/windows.html [49, 50] or with simple or partial correlations with 0.05 or lower. Correlations are considered significant at q = 0.05 but for exploratory purposes include those P values with 0.05 or lower. Correlations were carried out within each season and wintering method. For *Nosema*, the correlations between season (fall, winter and spring) and sampling location (brood area and entrance), were also analyzed using PROC CORR (SAS 1999).

Finally, a log-linear regression was carried out on fall and spring pathogens using pooled averages of brood and entrance samples. Data were analyzed by binary-logistic regression for each virus, using DataDesk 6·0 (Data Description, Inc [51] Ithaca, New York, USA). Seventy-five hives were separated into two groups live = 0 or dead = 1, based on the observations taken in spring at the end of the experiment.

The effect of fall treatment with fumagillin on *Nosema* spore counts in spring (pooled brood area and entrance) was compared with hives not treated with fumagillin within indoor and outdoor-wintered colonies by ANOVA using PROC MIXED (SAS 1999). Since fumagillin was applied to individual hives, hives were treated as replicates for this analysis.

## Results

### Change in colony size over winter

**Fig 1:**
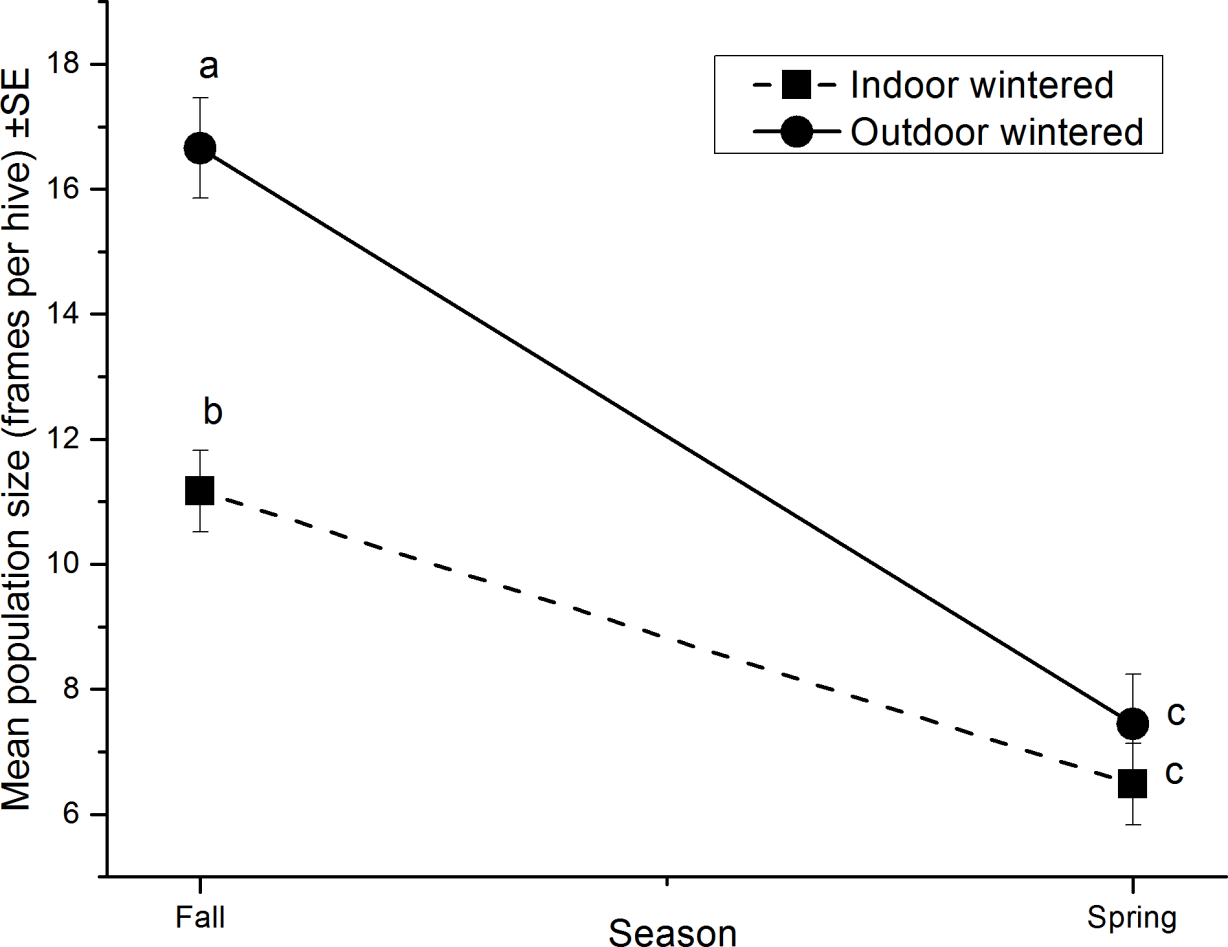
Change in colony population score (frames of bees, 1 frame = ~ 2,430 bees) from fall to spring, for indoor (-■-) and outdoor (─●─) wintered colonies. Means followed by the same letter are not significantly different (P > 0.05; Bonferroni).

The relative change in bee population size from fall to spring was affected by wintering method as indicated by a significant wintering method *season interaction (F = 138.12, df = 3, 72 P < 0.0001; Fig 1). Over the winter, the outdoor-wintered colonies suffered significantly higher bee population loss (55%) than indoor-wintered ones (42%). In fall, outdoor-wintered colonies had higher populations than indoor-wintered colonies (P < 0.05, SLICE) but by spring colony populations for outdoor-wintered colonies and indoor were similar (P<0.05, SLICE) (Fig 1). Overall, winter colony mortality (colonies that were dead after removal of winter wraps in spring) was 20% and did not differ with wintering method (df = 1, χ^2^ = 0.70, P = 0.40).

### Prevalence of honey bee pathogens and parasites

There was a significant interaction between season*pathogen—parasite (χ^2^ = 36.81, df = 9, P < 0.0001) therefore separate analyses were performed on each pathogen and parasites. Prevalence of parasites and pathogens was similar in each wintering method (χ^2^ = 2.72, df = 1, P = 0.10). *Varroa* were detected in 56–83% of colonies, but there was no difference in proportion of colonies with detectable mites among wintering methods (χ^2^ = 1.26, df = 1, P = 0.26) and among season (χ^2^ = 0.25, df = 1, P = 0.61) (Table 1). The honey bee tracheal mite was found in a low percentage of colonies (3% to 10%) and prevalence also did not vary with wintering method (χ^2^ = 0.14, df = 1, P = 0.70) or by season (χ^2^ = 1.29, df = 1, P = 0.25). *Nosema* spore prevalence increased from fall to spring seasons (χ^2^ = 15.97, df = 1, P < 0.0001) and within indoor-wintered colonies *Nosema* prevalence was higher in mid-winter than fall but remained at similar prevalence between mid-winter and spring (Table 1).

**Table 1:** Proportion of colonies (in two wintering methods) with detectable levels of parasites or pathogens.

Wintering method	Season	Number of colonies	*Varroa* (%)	Tracheal mite (%)	Nosema* (%) #	DWV (%)	BQCV (%)	SBV* (%) #	IAPV* (%)	KBV* (%)	CBPV (%)	ABPV (%)
Indoor	**Fall**	45	69	4	53a	100	98	40a	13	11	13	2
	**Mid-winter**	45	56	9	87b	87	82	84b	33	18	13	16
	**Spring**	40	58	10	90b	98	88	73b	20	20	13	10
Outdoor	Fall	30	83	3	47	100	100	50	13	13	10	3
	**Spring**	26	73	8	96	96	92	85	50	46	35	8
Pooled	**Fall**	75	76	3.5	50	100	99	45	13	12	11.5	2.5
Pooled	**Spring**	66	65.5	9	93	97	90	79	35	33	24	9

The levels of parasites or pathogens as measured by the alcohol wash method for *Varroa* on adult bees collected in the brood area (250 bees) and from colony entrances (200 bees approximately), spore count for *Nosema* spp (from 100 bees), thoracic slice method for tracheal mite (100 bees), and RT-PCR for viruses using 50 bees for brood area and 10 bees for entrance samples.

* = Overall seasonal change in prevalence (see results for statistics) from fall to spring averaged over both wintering methods.

**# =** Comparisons within indoor-wintered colonies for changes in seasonal prevalence (see results for statistics) proportions followed by the same letter within indoor-wintering hives are not significantly different (P > 0.05).

DWV = deformed wing virus; BQCV = black queen cell virus; SBV = sacbrood virus; IAPV = Israeli acute paralysis virus; KBV = Kashmir bee virus; CBPV = Chronic bee paralysis virus; ABPV = acute bee paralysis virus.

DWV and BQCV had the highest prevalence and were detected at similar frequencies in indoor and outdoor-wintered colonies (Table 1). Their prevalence did not change over winter (Table 1). Over both wintering methods, SBV prevalence increased from fall to spring (χ^2^ = 11.08, df = 1, P < 0.0009) and for indoor-wintered colonies (also sampled in mid-winter) prevalence increased from fall to mid-winter but remained at similar prevalence from mid-winter to spring. IAPV and KBV prevalence both increased over winter when averaged over both wintering methods (IAPV df = 1, χ^2^ = 7.97, P < 0.005, KBV df = 1, χ^2^ = 8.03, P < 0.005) but did not increase in prevalence from fall to mid-winter for indoor wintered colonies. CBPV and ABPV were detected in a comparatively low proportion of colonies and prevalence remained low the following spring (Table 1).

### Mean abundance of pathogens and parasites

The analyses for effects of wintering method (indoor and outdoor), season and location of sampling within the colony (entrance vs. brood area) on the mean abundance of parasites and pathogens are summarized in Table B in S1 File.

**Fig 2:**
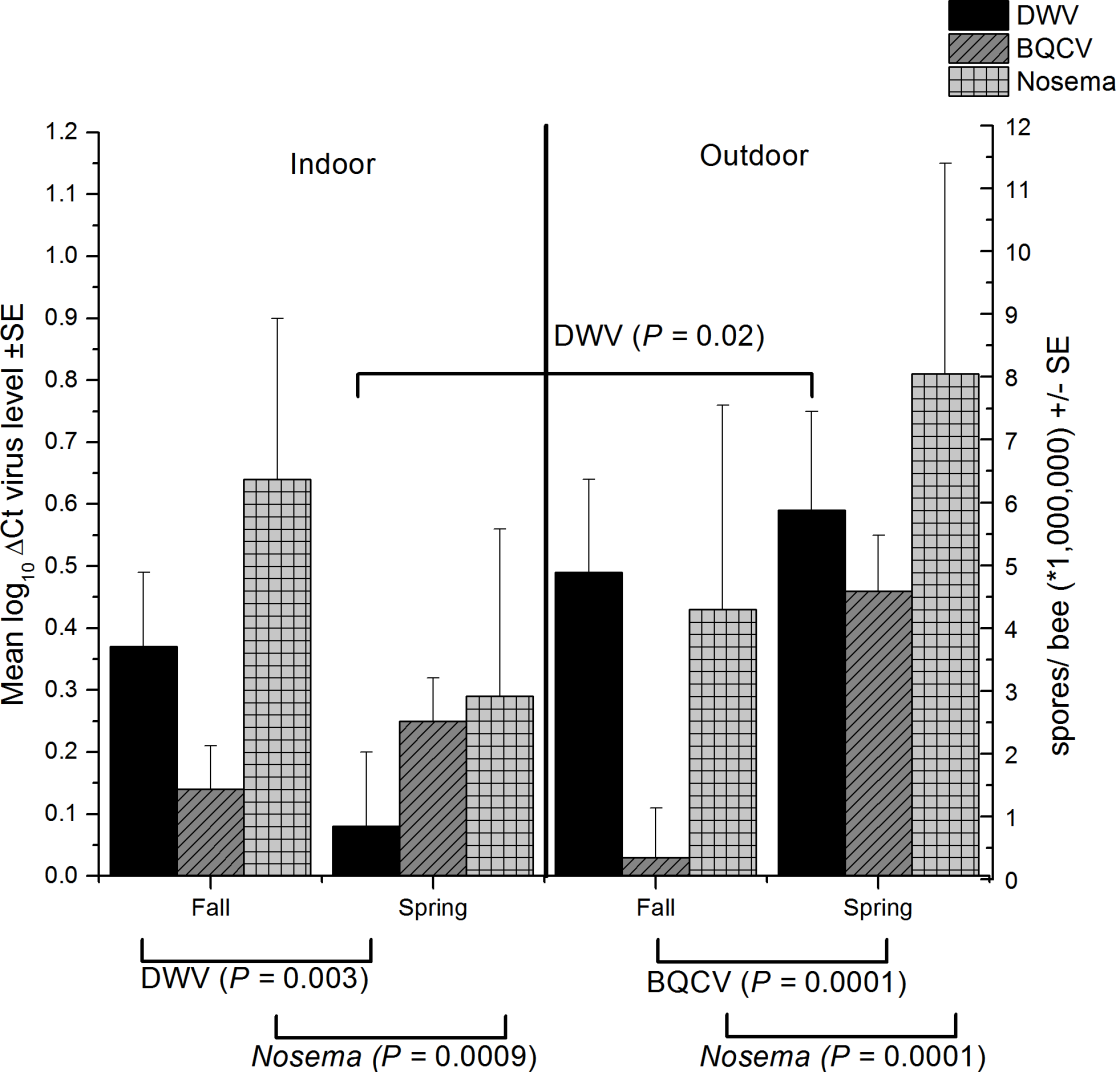
**Interactions between season (spring and fall) and wintering method (indoor and outdoor) (see Table B in S1 File) for DWV and BQCV concentrations (left axis) and mean abundance of *Nosema* (right axis) (± standard error).** Results of significant slices for each virus are indicated above the bars for slices by wintering method and below the graph for slices by season, (horizontal lines indicate significant slices). Data represent pooled values for brood area and entrance samples.

**Fig 3:**
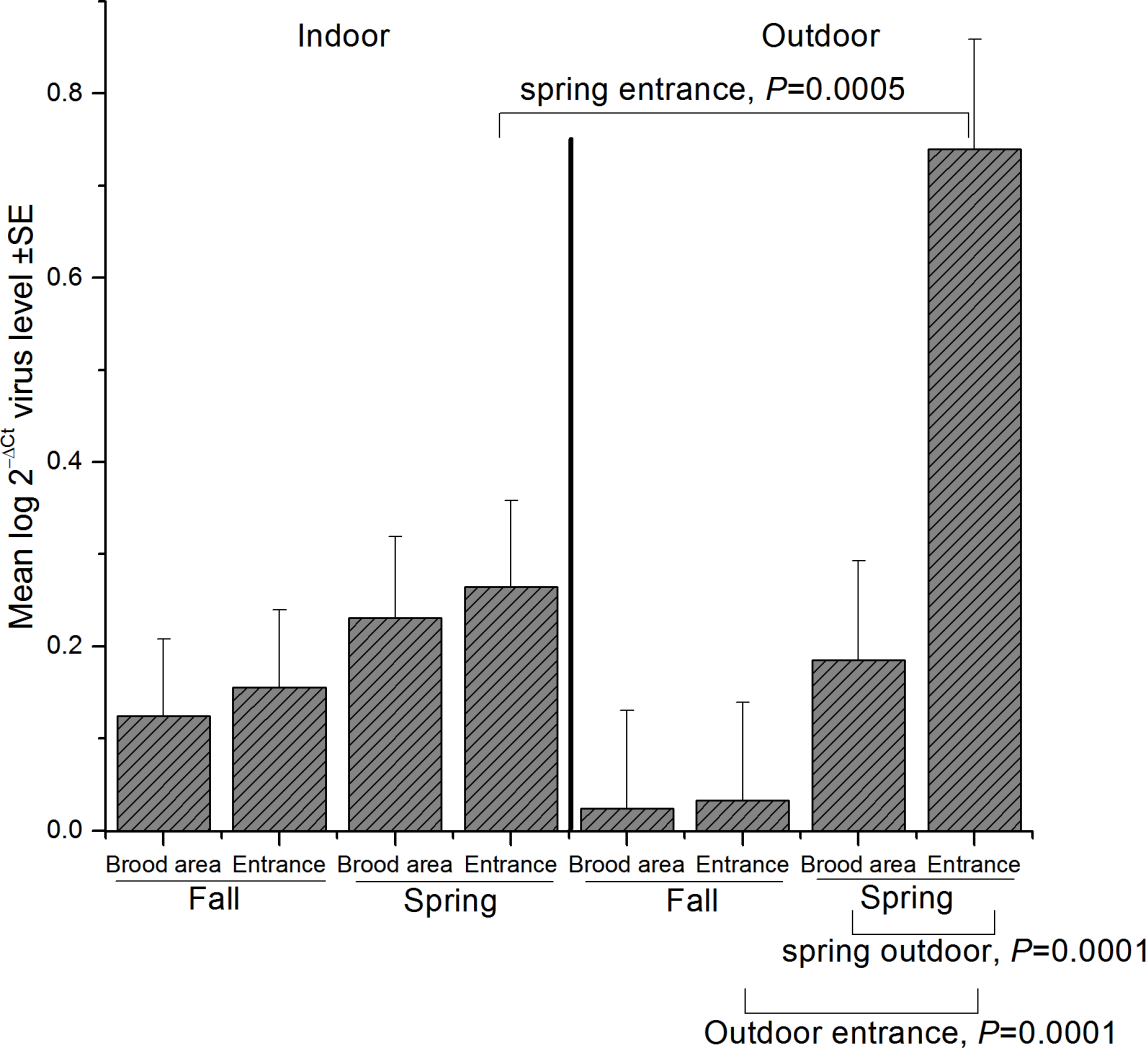
Interaction between season (spring and fall), wintering method (indoor and outdoor), and sampling location (brood area and entrance) (see Table B in S1 File) for BQCV concentrations (± standard error). Results of significant slices are indicated above the bars for wintering method and below the graph for season and wintering method, (horizontal lines indicate significant slices).

For *Nosema*, DWV and BQCV, there were significant interactions between season and wintering method (Table B in S1 File) although for BQCV the three-way interaction between season, wintering method and sampling location was also significant. In fall, nosema, DWV and BQCV all had similar levels in indoor- and outdoor-wintered hives (P > 0.05 Slice) (Figs 2 and 3). The mean abundance of *Nosema* (millions of spores / bee) decreased from fall to spring in indoor-wintered colonies (F = 11.59, df = 1, 129, P < 0.0009) but increased from fall to spring in outdoor-wintered colonies (F = 29.48, df = 1, 129, P < 0.0001 (Fig 2).

DWV concentration decreased from fall to spring in indoor-wintered colonies (F = 9.06, df = 1, 121, P < 0.003) but for outdoor wintered colonies DWV concentration was similar in spring and fall (P > 0.05 Slice). In spring DWV concentration was lower in indoor-wintered colonies (F = 5.07, df = 1, 121, P < 0.02), than outdoor-wintered colonies (P > 0.05 Slice). The wintering method*sampling method interaction was not significant (Table B in S1 File).

Significant season*wintering method*sampling location interactions were found only for BQCV (see Table B in S1 File). Overall, spring BQCV concentration was higher than fall (Fig 2) (F = 26.55, df = 1, 121, P < 0.0001) but the seasonal differences that occurred in the outdoor wintered colonies were found only in entrance collected bees (F = 29.61, df = 1, 121, P < 0.0001). Fall BQCV levels were similar in indoor and outdoor-wintered colonies for both brood area and entrance samples (Fig 3), (P > 0.05 Slice). Spring levels of BQCV were higher in outdoor-wintered hives than in indoor-wintered hives but only for entrance samples (F = 6.42, df = 3, 121, P < 0.0005). In outdoor-wintered colonies in spring, the virus levels in entrance samples were much higher than in brood area samples (F = 11.83, df = 3, 121, P < 0.0001). In outdoor-wintered colonies, the fall hive entrance samples of bees had much lower BQCV levels than the spring entrance samples (F = 14.15, df = 3, 121, P < 0.0001) (Fig 3).

**Fig 4:**
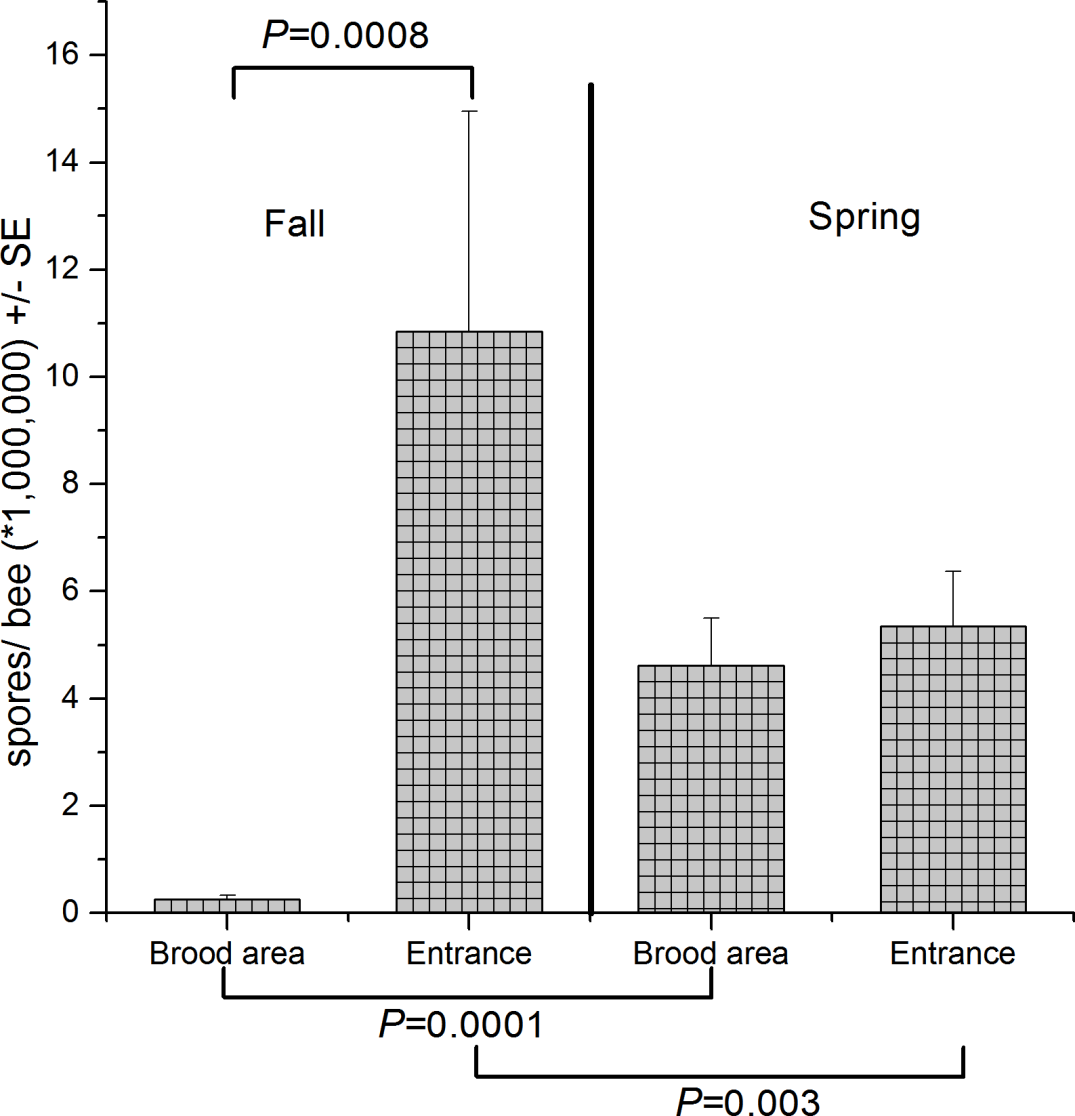
Interaction between season (fall and spring) and sampling location (brood area and entrance) (see Table B in S1 File) on mean abundance of *Nosema* (± standard error). Results of slices are indicated above the bars for sampling location and below the bars for season, (horizontal lines indicate significant slices). Data represent pooled values for indoor and outdoor samples.

For *Nosema* there was also a significant season*sampling location interaction (Table B in S1 File). *Nosema* levels were higher in entrance than brood area samples in fall (F = 11.91, df = 1, 129, P < 0.0008). However, no difference was seen between the two sampling locations in spring samples (P > 0.05, Slice). Brood area samples showed an increase in *Nosema* levels from fall to spring (F = 35.60, df = 1, 129, P < 0.0001), but entrance samples had higher *Nosema* levels in fall than in spring (F = 9.34 df = 1, 129, P < 0.003) (Fig 4).

**Fig 5:**
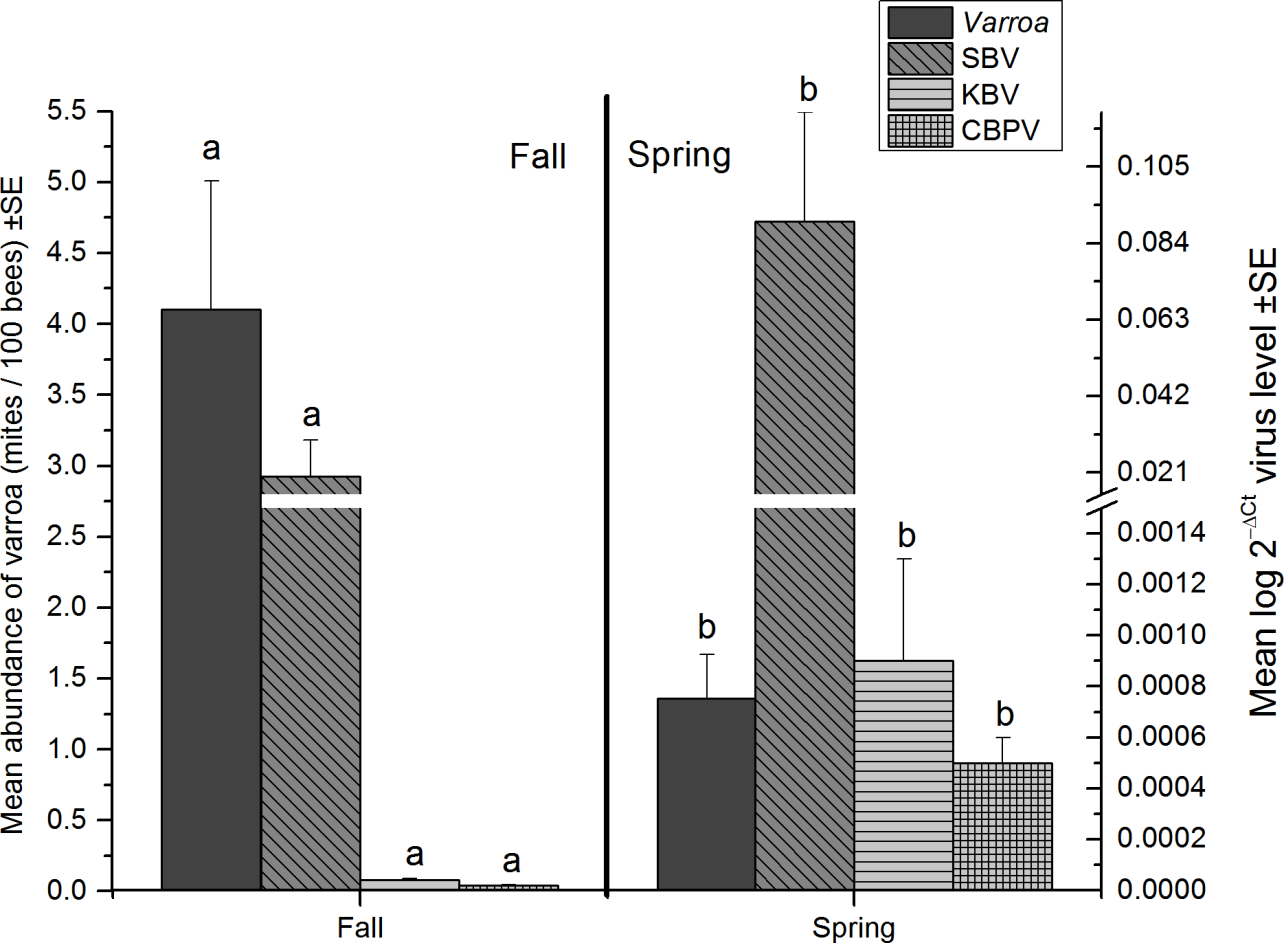
**Effect of season (fall and spring) on mean abundance of *Varroa* (left axis) and concentrations of viruses (SBV, KBV and CBPV) (right axis) (± standard error) (see Table B in S1 File).** Data represent pooled levels for both wintering methods (indoor and outdoor) and both sampling locations (brood area and entrance). Means followed by the same letter within a parasite or pathogen group are not significantly different (P > 0.05, Slice).

*Varroa* mean abundance (mites per 100 bees) was higher in fall than spring (Fig 5, Table B in S1 File) but did not show any interactions with wintering method or sample location. Although, overall *Varroa* levels were low, mean abundance in individual hives ranged from 0 to 52.6% for indoor-wintered hives and 0 to 24.4% for outdoor wintered hives. In spring, *Varroa* ranged from 0 to 9.8% for indoor-wintered hives and 0 to 13.1% for outdoor wintered hives. The concentration of SBV, KBV, and CBPV also changed with season (Table B in S1 File), the seasonal patterns for each of these viruses were lower in fall and increased in spring (Fig 5), but seasonal patterns for these viruses did not vary with wintering method or sample location (Table B in S1 File).

### Correlation analyses

Correlations between parasites, pathogens, honey bee population loss and spring population size were examined using simple and partial correlations and showed different patterns for indoor and outdoor-wintered hives. Correlations with a *q*-value of less than 0.2 are included in Table 2. For indoor-wintered colonies, partial correlations showed that only IAPV was negatively correlated with spring population size (*q* = 0.01) (Table 2A). There were significant correlations between several combinations of virus pairs for indoor wintered hives. Positive simple and partial correlations were found between SBV and KBV (*q* = 0.003) and also between IAPV and CBPV (*q* = 0.003). Simple correlations showed IAPV was correlated with KBV (*q* = 0.004) but partial correlations between IAPV and KBV were not significant (*q* = 0.06).

**Table 2:** Simple and partial correlations (Pearson’s) between colony parasite and pathogens for each category.

A Fall Indoor (N = 45)—Indoor-wintering							
Simple correlations				Partial correlations			
Variable pair	R	P- value	*q*-value	Variable pair	R	P- value	*q*-value
**SBV-KBV**	**+0.56**	**0.0001**	0.003*	**Pop-IAPV**	**-0.50**	**0.008**	0.01*
**IAPV-KBV**	**+0.53**	**0.0002**	0.004*	**SBV-KBV**	**+0.56**	**0.0001**	0.003*
**IAPV-CBPV**	**+0.63**	**0.0001**	0.003*	**IAPV-CBPV**	**+0.61**	**0.0001**	0.003*
**B Fall Outdoor (N = 30)—Outdoor-wintering**							
***Varroa* -DWV**	**+0.70**	**0.0001**	0.006*	***Varroa* -DWV**	**+0.71**	**0.0001**	0.006*
				***Nosema-*DWV**	**+0.58**	**0.002**	0.04*
**C Spring Indoor (N = 41)—Indoor wintering**							
**IAPV-ABPV**	**+1.00**	**0.0001**	0.003*	**IAPV-ABPV**	**+1.00**	**0.0001**	0.003*
**KBV-CBPV**	**+0.81**	**0.0001**	0.003*	**KBV-CBPV**	**+0.80**	**0.0001**	0.003*
***Nosema*-BQCV**	**+0.47**	**0.002**	0.03*				
**D Spring outdoor (N = 29)—Outdoor-wintering**							
***Varroa*–DWV**	**+0.53**	**0.003**	0.01*	**Bee loss-*Varroa***	**+0.61**	**0.0008**	0.006*
**DWV-KBV**	**+0.65**	**0.0002**	0.001*	**Bee loss- DWV**	**+0.59**	**0.001**	0.008*
**DWV-CBPV**	**+0.78**	**0.0001**	0.0007*	Bee loss-CBPV	+0.41	0.04	0.12
**BQCV-KBV**	**+0.45**	**0.01**	0.03*	**Pop-*Varroa***	**-0.55**	**0.003**	0.02*
**IAPV-ABPV**	**+0.72**	**0.0001**	0.0007*	**Pop-DWV**	**-0.71**	**0.0001**	0.001*
**KBV-CBPV**	**+0.81**	**0.0001**	0.0007*	**DWV-KBV**	**+0.63**	**0.0005**	0.005*
***Nosema*-BQCV**	**+0.50**	**0.006**	0.02*	**DWV-CBPV**	**+0.71**	**0.0001**	0.001*
				**BQCV-KBV**	**+0.55**	**0.004**	0.02*
				**IAPV-ABPV**	**+0.77**	**0.0001**	0.001*
				**KBV-CBPV**	**+0.79**	**0.0001**	0.001*
				***Nosema*-BQCV**	**+0.61**	**0.001**	0.006*

The number of colonies for each category is shown in parentheses. The Parasite (mean abundance of *Varroa* and prevalence of tracheal mite) and pathogen (mean abundance of *Nosema*, log 2^-ΔCt^ of DWV, BQCV, IAPV, SBV, KBV, CBPV and ABPV) levels, percent bee loss over winter and spring colony population size across all samples in fall and spring for indoor and outdoor wintering colonies. Brood area and entrance samples were pooled prior to analysis. Significant correlations are in boldface (q < 0.05). R = correlation coefficient. * = *q*-values of 0.05 or lower are considered significant. P-value = Pearson’s simple and partial correlations. *q*-value = FDR adjusted *q*-values with cut off of 0.2. DWV = deformed wing virus; BQCV = black queen cell virus; SBV = sacbrood virus; IAPV = Israeli acute paralysis virus; KBV = Kashmir bee virus; CBPV = Chronic bee paralysis virus; ABPV = acute bee paralysis virus.

Patterns were different for outdoor-wintered hives in fall. None of the variables were significantly correlated with either bee loss or spring population size after adjustments for false discovery rate were done (Table 2B). However, in outdoor-wintered colonies, positive simple and partial correlations were found between *Varroa* and DWV (*q* = 0.006) and partial correlations showed *Nosema* was also positively correlated with DWV (*q* = 0.04).

Correlation analyses were also performed on parasite and pathogen levels in colonies that survived the winter and these data also showed different patterns for indoor- and outdoor-wintering. In spring indoor-wintered colonies, no significant correlations were found between any parasites or pathogens and bee loss (Table 2C). However, simple correlations were found between *Nosema* and BQCV (*q* = 0.03). In spring, both simple and partial correlations showed IAPV levels were highly correlated with ABPV both in indoor and outdoor wintered colonies. Similarly, KBV levels were positively correlated with CBPV both in indoor and outdoor wintered colonies (Table 2C and 2D).

For outdoor wintered colonies, partial correlations showed both *Varroa* (*q* = 0.006) DWV were positively correlated with bee loss (*q* = 0.008) and negatively correlated with spring population size (*Varroa q* = 0.02; DWV *q* = 0.001) (Table 2D). For outdoor-wintered colonies in spring, both simple and partial correlations showed DWV levels were positively correlated with KBV (*q* = 0.001 simple; *q* = 0.005 partial) and CBPV (*q* = 0.0007 simple; *q* = 0.001 partial) in contrast to indoor-wintered hives where they were not. For outdoor-wintered colonies, both simple (*q* = 0.02) and partial (*q* = 0.006) showed *Nosema* levels were positively correlated with BQCV.

### Seasonal *Nosema* correlations

Mean abundance of *Nosema* from each of the two sample locations within the hive (brood area or entrance) in fall was correlated with levels in colonies in mid-winter and spring and with each other to assess which sample location would result in a better prediction of future *Nosema* levels. There was a weak, but positive correlation between fall *Nosema* level of entrance-collected bees and fall *Nosema* level of brood area-collected bees (Table C in S1 File). However, fall entrance samples were better predictors of mid-winter *Nosema* levels than brood area samples. Fall entrance and brood area *Nosema* levels both showed weak negative correlations with spring *Nosema* spore levels, but fall brood area samples were marginally better at predicting spring brood area levels than were the fall entrance-collected samples. Mid-winter *Nosema* levels were highly correlated with the spring brood area samples but only weakly correlated with spring entrance samples (Table C in S1 File). Interestingly, neither fall entrance-collected nor brood area-collected *Nosema* levels were correlated with spring entrance-collected levels.

### Binary logistic regression

Multivariate analysis of colonies sampled in fall and spring for parasites and pathogens were related to the proportion of dead colonies using (binary logistic regression for colonies ranked = dead = 1 or live = 0). Analysis of fall sampling parameters showed that the proportion of dead colonies increases with increasing DWV (F = 5.68, df = 1, 67, p < 0.02). Analysis of spring sampling parameters showed that the proportion of dead colonies increases with increasing SBV (F = 7.32, df = 1, 67, p < 0.009). No significant correlations were observed with other groups of pathogens and colony death.

### Effect of fumagillin treatment

Fall fumagillin treatment suppressed *Nosema* for indoor-wintered colonies (df = 1, 39 F = 15.17, P < 0.0003) with workers from colonies treated with fumagillin having lower mean abundance of Nosema (2.34 ± 0.91 SE million spores per bee) than untreated ones (8.24 ± 1.50 million spores per bee). Numerically, outdoor wintered, colonies showed a similar trend with lower spore counts in colonies treated with fumagillin (1.62 ± 2.31 million spores), than untreated colonies (8.86 ± 1.94 million spores), but the difference in suppression was not significant (df = 1, 28 F = 3.64, P > 0.07).

## Discussion

In this study we compared beekeeper-managed colonies across a broad geographic scale in two distinct environments: a “mild” stable environment (for colonies wintered indoors) and a “harsh” fluctuating environment (for colonies wintered outdoors) [38]. We sampled adult bees from each colony before (two locations), during (one location for indoor-wintered colonies only), and after (two locations) winter to characterize the effects of two parasitic mites, seven RNA viruses, and *Nosema* on honey bee colony mortality and population loss over winter. The results showed that outdoor-wintered colonies had greater relative reductions in bee population scores over winter than indoor-wintered colonies despite having a similar composition and level of parasites and pathogens prior to winter. Two viruses (DWV and BQCV) and one pathogen (*Nosema*) showed different seasonal patterns in indoor and outdoor-wintered colonies. Combinations of parasite and pathogen variables that were correlated with bee loss or spring size and each other also differed in the two wintering systems. Sample location affected assessment of *Nosema* and BQCV levels but did not affect assessment of other parasites or pathogens and this has implications for sampling to predict impacts of pathogens on colony loss.

Since 2006, honey bee winter colony losses in Canada have often exceeded 29% of the national total [1, 52] and have been at levels equivalent to levels of colony losses found in the U.S. and Europe [3]. In this broad scale study, overall death of colonies wintered in different environments was similar (20% loss) but the change in bee population score over winter was significantly greater in the outdoor-wintered colonies than in the indoor-wintered colonies. This was not likely a result of beekeeper management designed to achieve a specific spring population size. In northern regions, beekeepers often winter larger colonies (double brood chambers) outdoors and smaller colonies (single brood chamber) indoors as occurred in this study. Thus, the larger fall populations found in outdoor-wintered colonies were expected. However, under this type of management, spring populations in healthy double brood chamber colonies would typically consist of 16,000–25,000 bees, whereas single brood chamber colonies would have lower populations (7,800–10,000 bees) [53]. This did not occur in this study where spring population size of the outdoor-wintered hives (mostly double brood chamber) and indoor-wintered hives (mostly single brood chamber) were similar.

The prevalence and concentrations of the suite of parasites and pathogens analyzed in this study were similar for the indoor and outdoor-wintered colonies prior to implementation of wintering management. No single parasite or pathogen was highly correlated with winter bee loss in either of the two wintering methods in our study. However, the interactions between parasites, pathogens, colony loss and spring population size were very different in each of the two wintering environments. Thus, the environment to which colonies were exposed, in combination with management practices of beekeepers, likely played a role in affecting the different interactions between parasites and pathogens that were observed. Indoor-wintered colonies were maintained in a comparatively mild, stable environment (5°C) under total darkness for the entire winter period (November to March). In contrast, colonies wintered outdoors in our study were exposed to temperatures that ranged from -33°C to 17°C and would have been exposed to daily (or periodical) temperature fluctuations of up to 23°C. Little is known about how environmental stressors interact with pathogen and parasite webs in honey bees at the colony level. Lab studies have shown that comparatively small variations in brood nest temperature (shifts from 30 to 33°) can influence the severity of viruses in developing bees [54]. Field studies have shown that wintering honey bees in the more stable environments within wintering buildings allows colonies to survive winter under higher infestation levels of *Varroa*, either alone or in various combinations with tracheal mite and *Nosema* or other stressors [8, 14, 33, 34, 36]. However, before this work, interactions with viruses in different wintering environments have not been examined.

In this study, most beekeepers (23 of 25) treated colonies for *Varroa*. Overall, the average *Varroa* levels in late fall were well below the fall economic threshold for *Varroa* of > 3% in early fall and >10% in late fall that can result in significant winter colony mortality in this region of Canada [7–9]. It is likely some samples were taken before residual effects of the acaricide treatments brought mite levels fully under control as *Varroa* levels decreased further before spring. However, despite the acaricide treatments, there were still a few colonies that were detected at the time of fall sampling that were well above treatment thresholds in both indoor and outdoor treatment groups. The mites in these colonies may have escaped treatment due to acaricide resistance, which is common in Canada [1] or may have immigrated to colonies through drifting or robbing bees. It should also be noted that some colonies could have been above the threshold prior to fall sampling but had low mite levels when samples were collected and processed for parasites and pathogens as a result of prior acaricide treatment. The extent to which this might have occurred could not be quantified, as beekeepers did not know their mite levels prior to treatment.

*Varroa* on its own has major impacts on colony survival. *Varroa* feeding activity directly affects adult worker bees by removing their hemolymph and depleting protein and lipid reserves, which shortens their life span [55]. Despite the relatively low mite levels in our study, there was a positive correlation between *Varroa* mean abundance and bee loss and negative correlation between *Varroa* mean abundance and spring population size for outdoor-wintered hives (when sampled in spring). Others have found high rates of winter bee loss caused by *Varroa*, but usually in association with much higher mite levels (>10 mites per 100 bees) [1, 2, 9, 16]. *Varroa* and honey bee tracheal mites are also known to interact synergistically in enhancing bee losses [13, 14], but tracheal mites did not have a significant effect on bee loss in this study.

It is not known if the dynamics of *Varroa* feeding and its role as a vector and activator of viruses would be substantially different in clusters of bees wintered indoors and those wintered outdoors. It is likely that lower levels of environmental “stress” associated with indoor wintering may have contributed to the different patterns that were observed. *Varroa* mites are effective vectors of many viruses and play a major role in activating DWV, KBV, ABPV, IAPV, SBV, and CBPV to pathogenic levels in honey bees [26, 56–60]. *Varroa* weaken the bee’s immune systems, making them more susceptible to viruses, and act as effective vectors to spread viruses within colonies [58, 61–63]. *Varroa* and DWV together affect storage lipoproteins (vitellogenin) necessary for winter survival [16] and affect immune system function [64–66]. DWV alone could also be damaging [67, 68]. DWV replicates in various tissues such as the fat body [69] and can replicate in immature and adult bees and increase bee mortality even in the absence of *Varroa* [68]. Of the viruses tested, only DWV was associated with bee loss and low spring population size. We did not find any other direct correlations between *Varroa* and other viruses, which are often linked to poor bee health in different regions.

*Varroa* was correlated with DWV for fall samples in outdoor-wintered colonies. The high *Varroa* populations that were in some of our colonies would likely increase the chance of transmission of DWV and increase the susceptibility of bees to the virus [58, 59, 70]. Others have shown that viruses such as DWV can remain at high levels even after *Varroa* has been removed by acaricide treatment. These interactions with viruses are now thought to be a major factor associated with colony loss [19]. This is also likely to have occurred in our study to some extent as the most of mites were controlled by acaricide treatments but could have been at high levels prior to treatment.

Reductions in DWV from fall to spring in indoor-wintered hives may have been the result of either lower *Varroa* levels found in spring than in fall, highly infected bees dying and being removed from the colony over winter [16] or possibly the result of population turnover in the colonies through brood rearing during in indoor-wintering during winter and early spring. The outdoor wintering environment seemed to favour “maintenance” of DWV. Possibly interactions with *Nosema* or other viruses played role in facilitating DWV maintenance in outdoor-wintered colonies. Reductions in *Varroa* from fall to spring were not likely involved since *Varroa* was also lower in spring than in fall, during indoor-wintering where virus concentration declined over winter. Perhaps greater stress associated with the outdoor wintering environment suppressed immune responses in the bees. It is also possible that the acaricides used by beekeepers to control *Varroa* levels influenced DWV replication [17]; however, the same types of acaricides were applied in both environments.

Spring DWV concentration was associated with winter bee loss and low spring population size for outdoor wintering thereby building upon the growing evidence implicating DWV as a cause of colony losses in many environmental and bee- management contexts [15, 16, 19, 67, 71, 72]. Although DWV appeared to affect bee loss in combination with *Varroa*, it also was correlated with other pathogens. For outdoor-wintered colonies in spring, DWV was positively correlated with KBV and CBPV. It is possible DWV may make bees more susceptible to other virus infections; however, this requires further study.

Previously, IAPV has been associated with CCD [20] and has been demonstrated to cause mortality in honey bees [73]. IAPV causes increased risk for colony collapse in association with *N*. *apis* [20], but associations with CCD are not always correlated with *Nosema* [71]. IAPV is not consistently linked with CCD-like symptoms [71]. In our study, the relationship between IAPV and spring population size also differed with the wintering environment. IAPV levels did not change with season but we did find marginal positive correlations between fall IAPV and bee loss (*q* = 0.06) and negative correlations between IAPV and spring population sizes of colonies (*q* = 0.01) for colonies wintered indoors. Interestingly, no correlations between IAPV and bee loss parameters were evident in spring. Cornman et al. [71] did not find a link between bee loss and IAPV, but sampled colonies only after declines associated with colony collapse had occurred. This may explain in part why IAPV was not linked with bee loss in their study. If we had sampled only in spring, we also would not have found any correlation between IAPV and bee loss. However, environmental influences may have also affected links between IAPV and bee loss as we saw no link with IAPV and bee loss-related parameters in outdoor-wintered hives. Colonies in the outdoor-wintering environment may have succumbed to the presence of other stressors before IAPV could exert any effects on mortality.

Interactions between IAPV and other pathogens also varied with the wintering environment. For indoor-wintered colonies, we found positive correlations between IAPV and the viruses KBV and CBPV in fall and between IAPV and ABPV in spring. Whereas, for outdoor-wintered colonies, IAPV was positively correlated with ABPV only in spring. CBPV concentration increased from fall to spring in both wintering methods. Partial correlations of CBPV were marginally correlated with spring bee loss (*q* = 0.06) but only in outdoor wintered hives. We also found KBV concentrations increased from fall to spring; however, KBV was not correlated with bee loss in our study. ABPV, KBV and IAPV are closely related viruses linked to poor bee health [72, 74] and are usually damaging with very low levels of virus particles [70]. Recently, Francis et al, [72] showed that combinations of ABPV, KBV and IAPV and DWV levels were very high in untreated colonies that died during winter, compared to acaricide-treated survivors. ABPV is wide spread in Europe, where colony prevalence ranges from 8% to 69% and it has been linked to colony death [21, 22, 74, 75]. However, we found low prevalence (up to 16%) of ABPV in colonies, very low concentrations relative to other viruses and no association with *Varroa* in our study. ABPV concentrations did not change from fall to spring.

Sac brood virus is generally thought of as a disease of immature bees (brood), and typically occurs at low levels in spring, peaks in mid-summer and declines in fall following natural brood cycles [26]. In adult bees, the virus is also at lower levels in fall [76] but little is known about the seasonal dynamics of this virus over winter. In our study, SBV prevalence increased dramatically from fall to spring and was also very high in colonies sampled in mid-winter even though little brood would be present in colonies at that time. SBV concentrations also were higher in spring than in fall. Sac brood was the only virus strongly associated with colony death and was also the only virus that had higher prevalence in unhealthy colonies from Manitoba than in healthy colonies across Canada [77, 78]. It is not known if SBV plays a direct role in colony death or if it is an opportunistic pathogen that is favored when colonies are succumbing to other stresses. However, SBV was not correlated with any other parasites or pathogens linked to bee losses. Cornman et al [71] found SBV is correlated with IAPV but only in colonies not expressing symptoms of CCD. Although IAPV was linked to low spring populations for indoor-wintered colonies in our studies, we did not see any correlations between SBV and IAPV. In indoor-wintering environments, fall SBV concentrations were correlated with KBV levels, but Cornman et al [71] did not find an association between SBV and KBV. Cornman et al, [71] also found that in non-CCD colonies with *Nosema*, SBV is correlated with ABPV and CBPV, but we did not find any relationship between SBV and any of the pathogens.

Although *Nosema* plays a role in honey bee colony losses in some European countries [79, 80] its role in contributing to colony losses is controversial [60]. Recent studies from Germany [10] and Switzerland [16], found that neither *N*. *ceranae* or *N*. *apis* affect colony loss but this conflicts with outcomes from studies in Spain, where *N*. *ceranae* is one of the key parasites in colony losses [79–81]. *Nosema* was not correlated with bee loss in this study in either environment, even though spore counts were well above the nominal threshold in all three seasons. Although unadjusted partial correlations in fall outdoor-wintered colonies suggested *Nosema* was negatively correlated with spring population size, the *q*-values were not significant (*q* = 0.2). Manipulative experiments where colonies are exposed to different levels of infection in the absence of fumagillin treatments are needed to clarify the true impact of this disease on winter colony survival.

Our study showed that *Nosema* mean abundance increased over winter for outdoor-wintered hives and decreased over winter for indoor-wintered hives. Williams et al, [33] also compared *Nosema* in indoor- and outdoor-wintering environments in a smaller study (3 beekeeping operations). Although they did not show an overall effect of wintering method on *Nosema* intensity, they did find higher levels of *Nosema* in outdoor-wintered colonies than in indoor-wintered colonies for one operation. Fall treatments of *Nosema* with fumagillin were carried out in all of their colonies. In our study, 11 of 15 producers that wintered indoor, and 4 of 10 producers that wintered outdoors treated with fumagillin in fall for control of *Nosema*. For producers using indoor-wintering environments, those fall treatments resulted in lower spring *Nosema* spore counts than in producers that did not treat hives. However, in producers with outdoor-wintered hives spring *Nosema* spore counts did not significantly differ between those who treated with fumagillin and those that did not. This suggest that observed differences in the seasonal pattern of *Nosema* spore abundance for the two wintering environments may have been partly the results of differences in the residual efficacy of fumagillin in the two environments or a greater capacity for *Nosema* spores to replicate in colonies that are wintered outdoors compared to those wintered indoor.

Significant partial correlations between *Nosema* and DWV were observed for outdoor-wintered colonies in fall. Since spring DWV was associated with bee loss over winter and low spring population size in outdoor-wintered hives, this suggests a synergistic interaction between these pathogens may be occurring. Since *Nosema* suppresses the immune system in workers [82], the higher levels of DWV that were observed in spring outdoor-wintered hives could be related to inability to effectively control *Nosema* in outdoor wintered hives. Although it should be noted that *Varroa* is also correlated with DWV, *Varroa* may also be a factor affecting DWV concentration, (see earlier discussion), this needs further study. *Nosema ceranae* also causes severe damage to the mid-gut epithelial cells [28]. Mid-gut damage might facilitate exchange of viral pathogens across the gut wall and into the haemolymph [83] but antagonistic interactions may also occur [84]. Similar to our study, Cornman et al, [71] found a correlation between DWV and *N*. *ceranae* in colonies showing CCD-like symptoms but not in non-CCD colonies. However, [84] found a negative correlation between DWV and *N*. *ceranae* in mid-gut cells of honey bees in laboratory cage studies and [85] found no evidence for synergistic correlations between DWV and *N*. *ceranae* in Hawaiian colonies. Environmental influences could explain some of the differences as other studies did not have their colonies exposed to long periods of confinement or “harsh” wintering conditions.

Significant positive correlations were found between *Nosema* and BQCV for both indoor and outdoor wintered colonies in spring. Partial correlations between *Nosema* and BQCV in spring were only significant for outdoor-wintered colonies. BQCV is commonly associated with *Nosema* and this combination of these pathogens is thought to cause greater mortality in bees than either on its own [86, 87]. However, we did not see significant correlations between *Nosema* and bee loss or spring population size in either wintering environment. Marginal negative correlations between spring population and BQCV (*q* = 0.08) suggest BQCV may play a role in contributing to lower bee population but partial correlations between BQCV and spring population were not significant.

Of the seven viruses, parasites and pathogens tested, sampling location affected mean abundance estimates of only *Nosema* and BQCV. Our data suggest that for fall and spring sampling for *Varroa*, DWV, SBV IAPV, KBV, ABPV, and CBPV sampling from either entrances or brood area would produce similar estimates of colony level mean abundances. For *Nosema*, fall spore counts were dramatically different in entrance and brood area samples and although entrance samples may not best represent the total pathogen load in colonies at the time of sampling or impacts on colonies, they were better predictors of future pathogen levels in mid-winter and fall than were brood area samples. *Nosema* spore counts were likely higher in entrance-bee samples because foraging bees caught at entrance tend to be older bees and older bees are more likely to be infested with higher *Nosema* spores [31, 81].

BQCV levels differed with season, sample location and wintering method, indicating care must be taken when sampling for this pathogen and extrapolating interpretation of results of other studies where single samples are taken from only one location in the hive. Our data suggest that in fall, BQCV could be sampled from either entrance or brood area samples for both in indoor and outdoor-wintered colonies and provide similar estimate of colony-level mean abundance. However, in spring, mean abundance of BQCV differed with sample location for outdoor wintered hives; thus, colonies should be sampled from a combination of entrance and brood area samples to give a more realistic estimate of colony level mean abundance. BQCV shows a seasonal pattern that was similar to *Nosema*, in that the seasonal response differed with wintering method. For BQCV there was no change in mean abundance from fall to spring for indoor-wintered colonies and increase in mean abundance from fall to spring outdoor-wintered colonies. This is not unexpected since *Nosema* and BQCV are often highly correlated, as they were in spring samples outdoor-wintered colonies [56, 76].

In conclusion, our study showed that colonies under similar initial parasite and pathogen loads experience lower rates of bee loss in indoor-wintering management than in outdoor-wintering management. This suggests producers should consider the use of indoor wintering as a management tool to reduce winter loss when. We showed that parasite and pathogen interactions and seasonal changes in mean abundance differed in the two different wintering environments. Fall IAPV level was negatively correlated with spring population but only for indoor wintered colonies. Spring *Varroa* and DWV levels were positively correlated with bee loss and negatively correlated with spring population but only for outdoor-wintered hives. SBV was the only virus significantly associated with colony death over winter for both wintering methods. Sampling location in the hive needs to be considered when interpreting the pathogen load of colonies for *Nosema* and BQCV and for estimating their impact on colony populations. For these pathogens, the best location for sampling differs between pathogens and seasons. Further experiments are urgently required to better predict bee population losses that result from the interaction of honey bee viruses and to develop management practices that will reduce their impact on colonies.

## Supporting Information

S1 Fig Map showing regions from which bee samples were collected. (DOCX)

S1 File **Supporting information for: Table A, Primers used for the used for PCR analysis and qPCR analysis for quantification. Table B, Effect of wintering method, regions, sampling location in the hive and season on the relative levels of parasites and pathogens in honey bee colonies. Table C, Pearson’s correlation analyses of *Nosema* levels in entrance and brood area samples collected in fall, mid-winter and spring for colonies wintered indoors**.
(DOCX)

## References

[1] Currie RW, Pernal SF, Guzman-Novoa E. Honey bee colony losses in Canada. J Apic Res. 2010;49(1):104–6. .

[2] Guzman-Novoa E, Eccles L, Calvete Y, McGowan J, Kelly PG, Correa-Benitez A. *Varroa destructor* is the main culprit for the death and reduced populations of overwintered honey bee (*Apis mellifera*) colonies in Ontario, Canada. Apidologie. 2010;41(4):443–50. 10.1051/apido/2009076 .

[3] vanEngelsdorp D, Meixner MD. A historical review of managed honey bee populations in Europe and the United States and the factors that may affect them. J Invertebr Pathol. 2010;103:580–95. 10.1016/j.jip.2009.06.011 . 19909973

[4] Chauzat P, Laurent M, Rivière M-P, Saugeon C, Hendrikx P, Ribière-Chabert M, et al A Pan-European Epidemiological Study on Honey Bee Colony Losses 2012–2013. European Union Reference Laboratory for Honeybee Health, Brussels, Rapport technique. 2014.

[5] vanEngelsdorp D, Evans JD, Saegerman C, Mullin C, Haubruge E, Nguyen BK, et al Colony Collapse Disorder: A descriptive study. PLoS one. 2009;4(8):e6481 ISI:000268637700007. 10.1371/journal.pone.0006481 19649264 PMC2715894

[6] Desai SD, Kumar S, Currie RW. Occurrence, detection, and quantification of economically important viruses in healthy and unhealthy honey bee (Hymenoptera: Apidae) colonies in Canada. The Canadian Entomologist. 2016;148(01):22–35. 10.4039/tce.2015.23

[7] Gatien P, Currie RW. Timing of acaricide treatments for control of low-level populations of *Varroa destructor* (Acari: Varroidae) and implications for colony performance of honey bees. The Canadian Entomologist. 2003;135:749–63.

[8] Currie RW. Economic threshold for Varroa on the Canadian prairies Canadian Association of Professional Apiculturists (CAPA) http://capabeesorg/content/uploads/2013/02/varroathresholdpdf. 2008.

[9] Currie RW, Gatien P. Timing acaricide treatments to prevent *Varroa destructor* (Acari: Varroidae) from causing economic damage to honey bee colonies. The Canadian Entomologist. 2006;138(2):238–52.

[10] Genersch E, von der Ohe W, Kaatz H, Schroeder A, Otten C, Buchler R, et al The German bee monitoring project: a long term study to understand periodically high winter losses of honey bee colonies. Apidologie. 2010;41(3):332–52. 10.1051/apido/2010014 .

[11] Schafer MO, Ritter W, Pettis JS, Neumann P. Winter losses of honeybee colonies (Hymenoptera: Apidae): the role of infestations with *Aethina tumida* (Coleoptera: Nitidulidae) and *Varroa destructor* (Parasitiformes: Varroidae). J Econ Entomol. 2010;103(1):10–6. 10.1603/ec09233 . 20214362

[12] van Dooremalen C, Gerritsen L, Cornelissen B, van der Steen JJM, van Langevelde F, Blacquière T. Winter survival of individual honey bees and honey bee colonies depends on level of *Varroa destructor* infestation. PLoS one. 2012;7(4):e36285 10.1371/journal.pone.0036285 22558421 PMC3338694

[13] Downey DL, Winston ML. Honey bee colony mortality and productivity with single and dual infestations of parasitic mite species. Apidologie. 2001;32(6):567–76.

[14] Currie RW. Management of parasitic mites in honey bees. Saskatchewan Agrifood Innovation Fund, Project.97000002. September 25, 2001 2001 Contract No.:.

[15] Dainat B, Evans JD, Chen YP, Gauthier L, Neumann P. Predictive markers of honey bee colony collapse. PLoS one. 2012;7(2):e32151 MEDLINE: 10.1371/journal.pone.0032151 22384162 PMC3285648

[16] Dainat B, Evans JD, Chen YP, Gauthier L, Neumann P. Dead or alive: deformed wing virus and *Varroa destructor* reduce the life span of winter honeybees. Appl Environ Microbiol. 2012;78(4):981–7. MEDLINE: 10.1128/AEM.06537-11 22179240 PMC3273028

[17] Locke B, Forsgren E, Fries I, de Miranda JR. Acaricide treatment affects viral dynamics in *Varroa destructor*-infested honey bee colonies via both host physiology and mite control. Appl Environ Microbiol. 2012;78(1):227–35. 10.1128/aem.06094-11 . 22020517 PMC3255632

[18] Hung ACF, Adams JR, Shimanuki H. Bee parasitic mite syndrome (II): the role of varroa mite and viruses. Am Bee J. 1995;135(10):702–4.

[19] Highfield AC, El Nagar A, Mackinder LCM, Noel L, Hall MJ, Martin SJ, et al Deformed wing virus implicated in overwintering honeybee colony losses. Appl Environ Microbiol. 2009;75(22):7212–20. 10.1128/aem.02227-09 . 19783750 PMC2786540

[20] Cox-Foster DL, Conlan S, Holmes EC, Palacios G, Evans JD, Moran NA, et al A metagenomic survey of microbes in honey bee colony collapse disorder. Science. 2007;318:283–7. 10.1126/science.1146498 . 17823314

[21] Berthoud H, Imdorf A, Haueter M, Radloff S, Neumann P. Virus infections and winter losses of honey bee colonies (*Apis mellifera*). J Apic Res. 2010;49(1):60–5. 10.3896/ibra.1.49.1.08 .

[22] Nguyen BK, Ribiere M, vanEngelsdorp D, Snoeck C, Saegerman C, Kalkstein AL, et al Effects of honey bee virus prevalence, *Varroa destructor* load and queen condition on honey bee colony survival over the winter in Belgium. J Apic Res. 2011;50(3):195–202. 10.3896/ibra.1.50.3.03 .

[23] Anderson DL, East IJ. The latest buzz about colony collapse disorder. Science. 2008;319(5864):724–5. . 10.1126/science.319.5864.724c 18258878

[24] Chen YP, Pettis JS, Collins A, Feldlaufer MF. Prevalence and transmission of honeybee viruses. Appl Environ Microbiol. 2006;72(1):606–11. 10.1128/aem.72.1.606-611.2006 . 16391097 PMC1352288

[25] Chen YP, Higgins JA, Feldlaufer MF. Quantitative real-time reverse transcription-PCR analysis of deformed wing virus infection in the honeybee (*Apis mellifera* L.). Appl Environ Microbiol. 2005;71(1):436–41. 10.1128/aem.71.1.436-441.2005 . 15640219 PMC544241

[26] Bailey L, Ball BV. Honey bee pathology 2nd ed: Academic Press, San Diego, CA; 1991.

[27] Fries I, Feng F, daSilva A, Slemenda SB, Pieniazek NJ. *Nosema ceranae* n. sp. (Microspora, Nosematidae), morphological and molecular characterization of a microsporidian parasite of the Asian honey bee *Apis cerana* (Hymenoptera, Apidae). Eur J Protistol. 1996;32(3):356–65. .

[28] Fries I. *Nosema ceranae* in European honey bees (*Apis mellifera*). J Invertebr Pathol. 2010;103:S73–S9. 10.1016/j.jip.2009.06.017 19909977

[29] Botías C, Martín-Hernández R, Barrios L, Meana A, Higes M. *Nosema* spp. infection and its negative effects on honey bees (*Apis mellifera iberiensis*) at the colony level. Veterinary research. 2013;44(1):1–15. 23574888 10.1186/1297-9716-44-25 PMC3640932

[30] Ostermann DJ, Currie RW. Effect of formic acid formulations on honey bee (Hymenoptera: Apidae) colonies and influence of colony and ambient conditions on formic acid concentration in the hive. J Econ Entomol. 2004;97(5):1500–8. 15568335 10.1603/0022-0493-97.5.1500

[31] Smart MD, Sheppard WS. *Nosema ceranae* in age cohorts of the western honey bee (*Apis mellifera*). J Invertebr Pathol. 2012;109(1):148–51. 10.1016/j.jip.2011.09.009 . 22001631

[32] Fries I, Chauzat M-P, Chen Y-P, Doublet V, Genersch E, Gisder S, et al Standard methods for *Nosema* research. J Apic Res. 2013;52(1):1–28.

[33] Williams GR, Shutler D, Rogers REL. Effects at Nearctic north-temperate latitudes of indoor versus outdoor overwintering on the microsporidium *Nosema ceranae* and western honey bees (*Apis mellifera*). J Invertebr Pathol. 2010;104(1):4–7. 10.1016/j.jip.2010.01.009 . 20123103

[34] Bahreini R, Currie R. Increasing the economic threshold for fall treatment of varroa mite (*Varroa destructor* A.&T.) in honey bees (*Apis mellifera* L.) by using mite resistant stocks in the prairie-region of Canada. Hive lights. 2009;22:11–22.

[35] Bahreini R, Currie R. Breeding bees resistant to parasites and diseases. Bee- IPM workshop, 8–9 Feb, 2011; Edmonton, Alberta2011.

[36] Bahreini R, Currie RW. Influence of honey bee genotype and wintering method on wintering performance of *Varroa destructor* (Parasitiformes: Varroidae)-infected honey bee (Hymenoptera: Apidae) colonies in a Northern climate. J Econ Entomol. 2015:1–11; 0.1093/jee/tov164. 10.1093/jee/tov164 26470288

[37] Bahreini R, Currie RW. The influence of *Nosema* (Microspora: Nosematidae) infection on honey bee (Hymenoptera: Apidae) defense against *Varroa destructor* (Mesostigmata: Varroidae). J Invertebr Pathol. 2015;132:57–65. 10.1016/j.jip.2015.07.019 26283465

[38] Currie RW, Dixon D, Tuckey K, van Westendorp P. Beekeeping in Western Canada (Gruszka J. Editor): Alberta Agriculture, Food and Rural Development, Edmonton, Alberta, Canada; 1998 1998.

[39] Burgett M, Burikam I. Number of adult honey bees (Hymenoptera: Apidae) occupying a comb: a standard for estimating colony populations. J Econ Entomol. 1985;78:1154–6.

[40] Bush A, Lafferty K, Lotz J, Shostak A. Parasitology meets ecology on its own terms: Margolis et al. revisited. The Journal of Parasitology. 1997;83(4):575–83. 9267395

[41] Delfinado-Baker M. *Acarapis woodi* in the United States. Am Bee J. 1984;124(11):805–6.

[42] Cantwell GE. Standard methods for counting *Nosema* spores. Am Bee J. 1970;110(6):222–3.

[43] Persico M, Capasso M, Persico E, Svelto M, Russo R, Spano D, et al Suppressor of cytokine signaling 3 (SOCS3) expression and hepatitis C virus-related chronic hepatitis: Insulin resistance and response to antiviral therapy. Hepatology. 2007;46(4):1009–15. 10.1002/hep.21782 . 17668875

[44] Snedecor G, Cochran W. Statistical methods: The Iowa State University Press, Ames, Iowa, USA; 1980.

[45] SAS. SAS/STAT user's Guide, SAS Institute Inc., Cary, North Carolina, USA 1999.

[46] Benjamini Y, Hochberg Y. Controlling the false discovery rate: a practical and powerful approach to multiple testing. Journal of the Royal Statistical Society Series B (Methodological). 1995;57(1):289–300.

[47] Storey JD, Tibshirani R. Statistical significance for genomewide studies. Proceedings of the National Academy of Sciences. 2003;100(16):9440–5. 10.1073/pnas.1530509100 PMC170937 12883005

[48] Parker R, Guarna MM, Melathopoulos AP, Moon K-M, White R, Huxter E, et al Correlation of proteome-wide changes with social immunity behaviors provides insight into resistance to the parasitic mite, *Varroa destructor*, in the honey bee (*Apis mellifera*). Genome Biology. 2012;13:R81 10.1186/gb-2012-13-9-r81 23021491 PMC3491398

[49] Storey JD. A direct approach to false discovery rates. Journal of the Royal Statistical Society: Series B (Statistical Methodology). 2002;64(3):479–98.

[50] Storey JD, Taylor JE, Siegmund D. Strong control, conservative point estimation and simultaneous conservative consistency of false discovery rates: a unified approach. Journal of the Royal Statistical Society: Series B (Statistical Methodology). 2004;66(1):187–205.

[51] Data Description. Inc. Data Desk: Statistics Guide, Data Description, Inc., Ithaca, New York 1992.

[52] Van der Zee R, Pisa L, Andonov S, Brodschneider R, Charrière J-D, Chlebo R, et al Managed honey bee colony losses in Canada, China, Europe, Israel and Turkey, for the winters of 2008–9 and 2009–10. J Apic Res. 2012;51(1):100–14.

[53] Nelson DL. Population dynamics of indoor wintered colonies. In Apimondia '99 Congress XXXVI; Vancouver, Canada. 1331999.

[54] Prisco GD, Zhang X, Pennacchio F, Caprio E, Li J, Evans JD, et al Dynamics of persistent and acute deformed wing virus infections in honey bees, *Apis mellifera*. Viruses. 2011;3(12):2425–41. 10.3390/v3122425 22355447 PMC3280512

[55] Weinberg KP, Madel G. The influence of the mite *Varroa jacobsoni* oud. on the protein concentration and the hemolymph volume of the brood of worker bees and drones of the honey bee *Apis mellifera* L. Apidologie. 1985;16(4):421–35. 10.1051/apido:19850407 .

[56] Berenyi O, Bakonyi T, Derakhshifar I, Koglberger H, Nowotny N. Occurrence of six honeybee viruses in diseased Austrian apiaries. Appl Environ Microbiol. 2006;72(4):2414–20. 10.1128/aem.72.4.2414-2420.2006 . 16597939 PMC1449027

[57] Shen MQ, Yang XL, Cox-Foster D, Cui LW. The role of varroa mites in infections of Kashmir bee virus (KBV) and deformed wing virus (DWV) in honey bees. Virology. 2005;342(1):141–9. 10.1016/j.virol.2005.07.012 . 16109435

[58] Yang XL, Cox-Foster D. Effects of parasitization by *Varroa destructor* on survivorship and physiological traits of *Apis mellifera* in correlation with viral incidence and microbial challenge. Parasitology. 2007;134(3):405–312. 17078903 10.1017/S0031182006000710

[59] Yang XL, Cox-Foster DL. Impact of an ectoparasite on the immunity and pathology of an invertebrate: Evidence for host immunosuppression and viral amplification. Proc Natl Acad Sci U S A. 2005;102(21):7470–5. 10.1073/pnas.0501860102 . 15897457 PMC1140434

[60] Le Conte Y, Ellis M, Ritter W. *Varroa* mites and honey bee health: can *Varroa* explain part of the colony losses? Apidologie. 2010;41(3):353–63.

[61] De Jong D. Mites: Varroa and other parasites of brood Morse RM, Flottum PK (Eds), Honey bees pests, predators, and diseases (3rd ed), The A I Root Company, Medina, OH 1997 p. 279–327.

[62] Amdam GV, Hartfelder K, Norberg K, Hagen A, Omholt SW. Altered physiology in worker honey bees (Hymenoptera: Apidae) infested with the mite *Varroa destructor* (Acari: Varroidae): a factor in colony loss during overwintering? J Econ Entomol. 2004;97(3):741–7. 15279246 10.1093/jee/97.3.741

[63] Kovac H, Crailsheim K. Lifespan of *Apis mellifera carnica* Pollm. infested by *Varroa jacobsoni* Oud. in relation to season and extent of infestation. J Apic Res. 1988;27(4):230–8.

[64] Nazzi F, Brown SP, Annoscia D, Del Piccolo F, Di Prisco G, Varricchio P, et al Synergistic parasite-pathogen interactions mediated by host immunity can drive the collapse of honeybee colonies. PLoS Pathogens. 2012;8(6):e1002735 10.1371/journal.ppat.1002735 22719246 PMC3375299

[65] Li Q, Verma IM. NF-kappaB regulation in the immune system. Nat Rev Immunol. 2002;2(10):725–34. 12360211 10.1038/nri910

[66] Ghosh S, Karin M. Missing pieces in the NF-B puzzle. Cell. 2002;109(2 Suppl 1):S81–S96. 11983155 10.1016/s0092-8674(02)00703-1

[67] Martin SJ, Highfield AC, Brettell L, Villalobos EM, Budge GE, Powell M, et al Global honey bee viral landscape altered by a parasitic mite. Science. 2012;336(6086):1304–6. 10.1126/science.1220941 22679096

[68] Desai SD, Eu YJ, Whyard S, Currie RW. Reduction in deformed wing virus infection in larval and adult honey bees (*Apis mellifera* L.) by double-stranded RNA ingestion. Insect Mol Biol. 2012;21(4):446–55. 10.1111/j.1365-2583.2012.01150.x 22690671

[69] Fievet J, Tentcheva D, Gauthier L, de Miranda JR, Cousserans F, Colin ME, et al Localization of deformed wing virus infection in queen and drone *Apis mellifera* L. Virology Journal. 2006;3:1–5. doi: 16 10.1186/1743-422x-3-16 PubMed PMID: ISI:000247495500001 16569216 PMC1475838

[70] Schroeder DC, Martin SJ. Deformed wing virus: The main suspect in unexplained honeybee deaths worldwide. Virulence. 2012;3(7):589–91. 10.4161/viru.22219 23154287 PMC3545936

[71] Cornman RS, Tarpy DR, Chen Y, Jeffreys L, Lopez D, Pettis JS, et al Pathogen webs in collapsing honey bee colonies. PLoS one. 2012;7(8):e43562 10.1371/journal.pone.0043562 22927991 PMC3424165

[72] Francis RM, Nielsen SL, Kryger P. Varroa-virus interaction in collapsing honey bee colonies. PLoS one. 2013;8(3):e57540 10.1371/journal.pone.0057540 23526946 PMC3602523

[73] Maori E, Lavi S, Mozes-Koch R, Gantman Y, Peretz Y, Edelbaum O, et al Isolation and characterization of Israeli acute paralysis virus, a dicistrovirus affecting honeybees in Israel: evidence for diversity due to intra- and inter-species recombination. J Gen Virol. 2007;88:3428–38. 10.1099/vir.0.83284-0 . 18024913

[74] de Miranda JR, Cordoni G, Budge G. The Acute bee paralysis virus-Kashmir bee virus-Israeli acute paralysis virus complex. J Invertebr Pathol. 2010;103:S30–S47. 10.1016/j.jip.2009.06.014 . 19909972

[75] Allen MF, Ball BV. The incidence and world distribution of honey bee viruses. Bee World. 1996;77(3):141–62. .

[76] Tentcheva D, Gauthier L, Zappulla N, Dainat B, Cousserans F, Colin ME, et al Prevalence and seasonal variations of six bee viruses in *Apis mellifera* L. and *Varroa destructor* mite populations in France. Appl Environ Microbiol. 2004;70(12):7185–91. 10.1128/aem.70.12.7185-7191.2004 . 15574916 PMC535170

[77] Desai S. The potential impact of pathogens on honey bee, *Apis mellifera* L., colonies and possibilities for their control Winnipeg: University of Manitoba; 2014.

[78] Desai SD, Kumar S, Currie RW. Occurrence, detection, and quantification of economically important viruses in healthy and unhealthy honey bee (Hymenoptera: Apidae) colonies in Canada. The Canadian Entomologist. 2015;FirstView(Supplement -1):1–14. 10.4039/tce.2015.23

[79] Higes M, Martin-Hernandez R, Garrido-Bailon E, Gonzalez-Porto AV, Garcia-Palencia P, Meana A, et al Honeybee colony collapse due to *Nosema ceranae* in professional apiaries. Environmental Microbiology Reports. 2009;1(2):110–3. 10.1111/j.1758-2229.2009.00014.x . 23765741

[80] Higes M, Martin-Hernandez R, Martinez-Salvador A, Garrido-Bailon E, Gonzalez-Porto AV, Meana A, et al A preliminary study of the epidemiological factors related to honey bee colony loss in Spain. Environmental Microbiology Reports. 2010;2(2):243–50. 10.1111/j.1758-2229.2009.00099.x . 23766075

[81] Higes M, Martin-Hernandez R, Botias C, Bailon EG, Gonzalez-Porto AV, Barrios L, et al How natural infection by *Nosema ceranae* causes honeybee colony collapse. Environmental Microbiology. 2008;10(10):2659–69. 10.1111/j.1462-2920.2008.01687.x . 18647336

[82] Antunez K, Martin-Hernandez R, Prieto L, Meana A, Zunino P, Higes M. Immune suppression in the honey bee (*Apis mellifera*) following infection by *Nosema ceranae* (Microsporidia). Environmental Microbiology. 2009;11(9):2284–90. 10.1111/j.1462-2920.2009.01953.x . 19737304

[83] Bromenshenk JJ, Henderson CB, Wick CH, Stanford MF, Zulich AW, Jabbour RE, et al Iridovirus and microsporidian linked to honey bee colony decline. PLoS one. 2010;5(10):e13181 10.1371/journal.pone.0013181 20949138 PMC2950847

[84] Costa C, Tanner G, Lodesani M, Maistrello L, Neumann P. Negative correlation between *Nosema ceranae* spore loads and deformed wing virus infection levels in adult honey bee workers. J Invertebr Pathol. 2011;108(3):224–5. 10.1016/j.jip.2011.08.012 21939664

[85] Martin SJ, Hardy J, Villalobos E, Martín-Hernández R, Nikaido S, Higes M. Do the honeybee pathogens *Nosema ceranae* and deformed wing virus act synergistically? Environmental Microbiology Reports. 2013; 10.1111/1758-2229.12052 PMC3806273 23864563

[86] Bailey L, Ball BV, Perry JN. Association of viruses with two protozoal pathogens of the honey bee. Ann Appl Biol. 1983;103(1):13–20. .

[87] Bailey L, Ball BV, Perry JN. Honeybee paralysis: Its natural spread and its diminished incidence in England and Wales. J Apic Res. 1983;22(3):191–5. .

