# Supplementary material for: Effects of Wintering Environment and Parasite–Pathogen Interactions on Honey Bee Colony Loss in North Temperate Regions
Source: PLoS One. 2016 Jul 22;11(7):e0159615. doi: 10.1371/journal.pone.0159615 (PMC4957765; doi:10.1371/journal.pone.0159615)
Supplement: S1 Fig — (DOCX) [file pone.0159615.s001.docx]

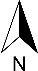


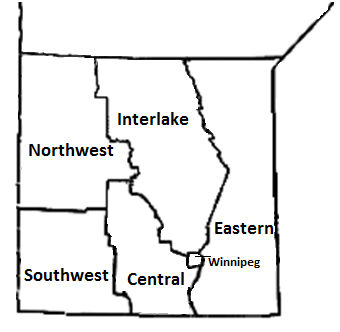

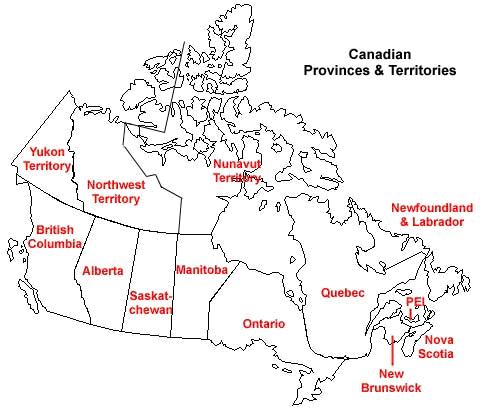


Scale for Manitoba

440 km

Fall sample collection Mid-winter sample collection Spring sample collection

| Sept | Oct | Nov | Dec | Jan | Feb | Mar | Apr | May |
| --- | --- | --- | --- | --- | --- | --- | --- | --- |

Cluster size Cluster size

Brood area bees Brood area bees Brood area bees

Entrance bees Entrance bees

S1 Fig. Map showing regions from which bee samples were collected. Dots represent the locations of samples collected from beekeepers (5 beekeepers per region). Below the sampling scheme is a time line indicating when fall, mid-winter and spring samples were taken. Samples taken at each sample date are listed below the bar.
