# Supplementary material for: Effects of Wintering Environment and Parasite–Pathogen Interactions on Honey Bee Colony Loss in North Temperate Regions
Source: PLoS One. 2016 Jul 22;11(7):e0159615. doi: 10.1371/journal.pone.0159615 (PMC4957765; doi:10.1371/journal.pone.0159615)
Supplement: S1 File — Supporting information for: Table A, Primers used for the used for PCR analysis and qPCR analysis for quantification. Table B, Effect of wintering method, regions, sampling location in the hive and season on the relative levels of parasites and pathogens in honey bee colonies. Table C, Pearson’s correlation analyses of Nosema levels in entrance and brood area samples collected in fall, mid-winter and spring for colonies wintered indoors. (DOCX) [file pone.0159615.s002.docx]

**Supporting Information for:**

**Effects of wintering environment and parasite–pathogen interactions on honey bee colony loss in North temperate regions.**

Suresh D. Desai* and Robert W. Currie

Department of Entomology, University of Manitoba, Winnipeg, Manitoba, Canada R3T 2N2

*Correspondence to: Suresh D. Desai**,** Department of Entomology, University of Manitoba, Winnipeg, Manitoba, Canada R3T 2N2, Tel: +1-2044746022, Fax: +1-2044747628, E-mail: suresh.desai@umanitoba.ca

**This file includes:**

Tables A to C

Supporting Information Reference List

Table A. Primers used for the used for PCR analysis and qPCR analysis for quantification.

| **Sl.No** | **Primers for bee viruses used in experiments** | **Product size (bp)** | **Reference** |
| --- | --- | --- | --- |
| 1 | RT-DWV-F: CGAAACCAACTTCTGAGGAA  RT-DWV-R: GTGTTGATCCCTGAGGCTTA | 174 | [1] |
| 2 | RT-BQCV-F: CCTGTATTCATGCATCTCAGA  RT-BQCV-R: GCAACAAGAAGAAACGTAAACCAC | 310 | Judy Chen (USDA) personal communication |
| 3 | RT-SBV-F: GTAGTCCAGTGCCCGATGTGT  RT-SBV-R: GCACCAAAAGTACCTCCCAA | 165 | Judy Chen (USDA) personal communication |
| 4 | RT-IAPV-F: GCGGAGAATATAAGGCTCAG  RT-IAPV-R: CTTGCAAGATAAGAAAGGGGG | 586 | [2] |
| 5 | RT-KBV1 F: GGTGTAGCAGCCATATTCG  RT-KBV1 R: CTCGCAAGTCTCCTAATTCG | 157 | This work |
| 6 | RT-CBPV F: TAAGTCGTTGTCGTATCAC  RT-CBPV R: AAGCAGTCATTCGTTATCC | 168 | This work |
| 7 | RT-ABPV-F: ATAACACGATGTTACCCGA  RT-ABPV-R: TCTTCTAAAGTACTGCGCTTG | 146 | Judy Chen (USDA) personal communication |
| 8 | β-actin-F: AGGAATGGAAGCTTGCGGTA  β-actin-R: AATTTTCATGGTGGATGGTGC | 181 | [3] |

DWV = deformed wing virus; BQCV = black queen cell virus; SBV = sacbrood virus; IAPV = Israeli acute paralysis virus; KBV = Kashmir bee virus; CBPV= Chronic bee paralysis virus; ABPV = acute bee paralysis virus.

**Table B.** Effect of wintering method, regions, sampling location in the hive and season on the relative levels of parasites and pathogens in honey bee colonies. Table S2 contd… (next page)

|  | Wintering method | | | |  | | Region | | | | Sample location | | | | | | Wintering method*  sampling location | | | |
| --- | --- | --- | --- | --- | --- | --- | --- | --- | --- | --- | --- | --- | --- | --- | --- | --- | --- | --- | --- | --- |
| Parameter | *F* | df | *P* |  | | *F* | | df | *P* |  | | *F* | df | *P* |  | *F* | | df | *P* |  |
| *Varroa* | 0.67 | 1, 19 | 0.42 |  | | 0.08 | | 4, 19 | 0.99 |  | | 2.24 | 1,64 | 0.14 |  | 0.06 | | 1, 64 | 0.81 |  |
| HBTM | 0.00 | 1, 19 | 0.97 |  | | 1.00 | | 4, 19 | 0.43 |  | | 0.00 | 1,65 | 0.99 |  | 0.07 | | 1, 65 | 0.79 |  |
| *Nosema* | 0.38 | 1, 19 | 0.54 |  | | 0.65 | | 4, 19 | 0.64 |  | | **7.31** | **1, 73** | **0.008** |  | 1.49 | | 1, 19 | 0.23 |  |
| DWV | 2.24 | 1, 19 | 0.15 |  | | 0.67 | | 4, 19 | 0.62 |  | | 0.63 | 1, 73 | 0.43 |  | 1.14 | | 1, 73 | 0.29 |  |
| BQCV | 0.04 | 1, 19 | 0.84 |  | | 1.98 | | 4, 19 | 0.14 |  | | **6.87** | **1, 73** | **0.01** |  | **4.27** | | **1, 73** | **0.04** |  |
| SBV | 0.11 | 1, 19 | 0.74 |  | | 1.02 | | 4, 19 | 0.42 |  | | 0.12 | 1, 73 | 0.73 |  | 0.05 | | 1, 73 | 0.83 |  |
| IAPV | 1.71 | 1, 19 | 0.21 |  | | 1.13 | | 4, 19 | 0.37 |  | | 0.03 | 1, 73 | 0.87 |  | 2.58 | | 1, 73 | 0.11 |  |
| KBV | 0.62 | 1, 19 | 0.44 |  | | 0.37 | | 4, 19 | 0.83 |  | | 1.81 | 1, 73 | 0.18 |  | 2.48 | | 1, 73 | 0.12 |  |
| CBPV | 4.29 | 1, 19 | 0.05* |  | | 0.87 | | 4, 19 | 0.50 |  | | 0.06 | 1, 73 | 0.80 |  | 0.74 | | 1, 73 | 0.39 |  |
| ABPV | 3.32 | 1, 19 | 0.08 |  | | 1.54 | | 4, 19 | 0.23 |  | | 0.95 | 1, 73 | 0.33 |  | 0.84 | | 1, 73 | 0.36 |  |

Significant correlations are in boldface. *F* = *F* value, df = degrees of freedom, *P* = P value, * P=0.052. HBTM= honey bee tracheal mite; DWV = deformed wing virus; BQCV = black queen cell virus; SBV = sacbrood virus; IAPV = Israeli acute paralysis virus; KBV = Kashmir bee virus; CBPV= Chronic bee paralysis virus; ABPV = acute bee paralysis virus.

**Table B.** contd…

|  | Season | | |  | Season* sampling location | | | | | |  | | Season * Wintering method | | | | | Season  *Wintering method*  sampling location | | |  |
| --- | --- | --- | --- | --- | --- | --- | --- | --- | --- | --- | --- | --- | --- | --- | --- | --- | --- | --- | --- | --- | --- |
| Parameter | *F* | df | *P* | | |  | *F* | df | *P* |  | | *F* | | df | *P* |  | *F* | | df | *P* | |
| *Varroa*† | **16.86** | **1, 65** | **0.0001** | | |  | - | - | - |  | | 0.01 | | 1, 65 | 0.91 |  | - | | - | - | |
| HBTM† | 0.35 | 1, 68 | 0.55 | | |  | - | - | - |  | | 0.51 | | 1, 68 | 0.48 |  | - | | - | - | |
| *Nosema* | **40.43** | **1,129** | **0.0001** | | |  | **4.08** | **1,129** | **0.045** |  | | **4.2** | | **1,129** | **0.04** |  | 0.00 | | 1,129 | 0.95 | |
| DWV | 1.61 | 1,121 | 0.21 | | |  | 2.17 | 1,121 | 0.14 |  | | **6.57** | | **1,121** | **0.01** |  | 0.03 | | 1,121 | 0.86 | |
| BQCV | **26.29** | **1,121** | **0.0001** | | |  | **5.90** | **1,121** | **0.02** |  | | **10.71** | | **1,121** | **0.001** |  | **5.8** | | **1,121** | **0.02** | |
| SBV | **8.65** | **1,120** | **0.003** | | |  | 1.19 | 1,120 | 0.27 |  | | 0.72 | | 1,120 | 0.39 |  | 0.84 | | 1,120 | 0.36 | |
| IAPV | 2.91 | 1,121 | 0.09 | | |  | 0.03 | 1,121 | 0.87 |  | | 0.04 | | 1,121 | 0.83 |  | 2.58 | | 1,121 | 0.11 | |
| KBV | **5.84** | **1,121** | **0.02** | | |  | 1.60 | 1,121 | 0.20 |  | | 1.59 | | 1,121 | 0.20 |  | 2.18 | | 1,121 | 0.14 | |
| CBPV | **16.38** | **1,121** | **0.0001** | | |  | 0.09 | 1,121 | 0.76 |  | | 3.59 | | 1,121 | 0.06 |  | 0.76 | | 1,121 | 0.39 | |
| ABPV | 1.31 | 1,121 | 0.26 | | |  | 0.99 | 1,121 | 0.32 |  | | 0.83 | | 1,121 | 0.36 |  | 0.87 | | 1,121 | 0.35 | |

Significant correlations are in boldface.† *Varroa* and honey bee tracheal mite were not sampled from entrances in fall. HBTM= honey bee tracheal mite; DWV = deformed wing virus; BQCV = black queen cell virus; SBV = sacbrood virus; IAPV = Israeli acute paralysis virus; KBV = Kashmir bee virus; CBPV= Chronic bee paralysis virus; ABPV = acute bee paralysis virus.

**Table C.** Pearson’s correlation analyses of *Nosema* levels in entrance and brood area samples collected in fall, mid-winter and spring for colonies wintered indoors.

| Parameter |  | Fall brood area | Winter | Spring entrance | Spring brood area |
| --- | --- | --- | --- | --- | --- |
| Fall entrance | R | **+0.37** | **+0.76** | +0.02 | **+0.30** |
|  | P- value | **0.001** | **0.0001** | 0.88 | **0.01** |
| Fall brood area | R |  | **+0.53** | +0.17 | **+0.42** |
|  | P- value |  | **0.0002** | 0.18 | **0.0004** |
| Winter | R |  |  | **+0.39** | **+0.71** |
|  | P- value |  |  | **0.02** | **0.0001** |
| Spring entrance | R |  |  |  | **+0.76** |
|  | P- value |  |  |  | **0.0001** |

Significant correlations are in boldface. R = correlation coefficient

1. Li J, Peng W, Wu J, Strange JP, Boncristiani H, Chen Y. Cross-species infection of deformed wing virus poses a new threat to pollinator conservation. J Econ Entomol. 2011;104(3):732-9.

2. Di Prisco G, Pennacchio F, Caprio E, Boncristiani HF, Jr., Evans JD, Chen Y. *Varroa destructor* is an effective vector of Israeli acute paralysis virus in the honeybee, *Apis mellifera*. J Gen Virol. 2011;92:151-5. doi: 10.1099/vir.0.023853-0. PubMed PMID: WOS:000286365200016.

3. Chen YP, Higgins JA, Feldlaufer MF. Quantitative real-time reverse transcription-PCR analysis of deformed wing virus infection in the honeybee (*Apis mellifera* L.). Appl Environ Microbiol. 2005;71(1):436-41. doi: 10.1128/aem.71.1.436-441.2005. PubMed PMID: ISI:000226458800055.
